# Lung cancer vaccines to enhance immune checkpoint inhibitor therapy: evidence and future perspectives

**DOI:** 10.1186/s13045-026-01778-7

**Published:** 2026-02-03

**Authors:** Zhiting Tang, Linjun Zha, Ruqiang Liang, Tianhong Li

**Affiliations:** 1 Division of Hematology and Medical Oncology, Department of Medicine, University of Missouri, Columbia, MO USA; 2 Division of Hematology and Oncology, Department of Internal Medicine, University of California Davis School of Medicine, University of California Davis Comprehensive Cancer Center, 4501 X Street, Suite 3016, Sacramento, CA USA; 3 Medial Service, Hematology and Oncology, Veterans Affairs Northern California Health Care System, Mather, CA USA

**Keywords:** Lung cancer, Cancer vaccines, Immune stimulants, Immune checkpoint inhibitors, ICI resistance, Neoantigens, Adaptive response, Immunological memory, Personalized neoantigen vaccine, Off-the-shelf vaccine

## Abstract

Immune checkpoint inhibitors (ICIs) have transformed the treatment landscape of lung cancer over the past decade, markedly improving antitumor responses, overall survival, and quality of life. However, durable clinical benefit is achieved in only a subset of patients, and resistance to ICIs remains a major clinical challenge. Mechanistically, resistance arises from multiple, often overlapping processes, including inadequate tumor antigen presentation, dysfunctional T-cell priming and expansion, and the presence of physical and immunosuppressive barriers within the tumor microenvironment that limit immune cell infiltration and effector function. Cancer vaccines have re-emerged as a rational immunotherapeutic strategy to overcome these obstacles by inducing *de novo* or amplifying pre-existing tumor-specific immune responses, thereby enhancing long-term immunological memory while maintaining a favorable safety profile. Advances in antigen discovery, neoantigen prediction, and vaccine platforms have accelerated the development of both personalized and off-the-shelf neoantigen vaccines. Although personalized neoantigen vaccines have gained considerable attention following the success of mRNA-based COVID-19 vaccines, off-the-shelf approaches offer advantages in scalability, cost, and manufacturing timelines, facilitating broader clinical implementation. Accumulating preclinical and clinical evidence suggests that cancer vaccines are more effective in the adjuvant setting than in the metastatic setting, where high tumor burden and an immunosuppressive tumor microenvironment constrain vaccine-induced immune responses. Consistent with their limited efficacy as monotherapy, contemporary clinical trials increasingly evaluate cancer vaccines in combination with ICIs or other immunotherapeutic agents to enhance T-cell activation, reverse immune suppression, and restore antitumor immunity. This review synthesizes current mechanistic insights, highlights ongoing clinical efforts, and discusses future directions for rational cancer vaccine development in lung cancer, with an emphasis on overcoming resistance to ICI.

## Introduction

Lung cancer remains the leading cause of cancer-related mortality both globally and in the United States [1, 2]. In 2022, lung cancer accounted for approximately 2.5 million new cases and 1.8 million deaths worldwide [1]. In the United States, an estimated 226,650 new diagnoses and 124,730 deaths are projected for 2025, representing 20.2% of all cancer-related fatalities [1, 2]. Lung cancer exhibits extensive histopathologic, molecular, genomic, and immunologic heterogeneity [3]. It is broadly classified into two main types: small cell lung cancer (SCLC) and non–small cell lung cancer (NSCLC), the latter comprises adenocarcinoma, squamous cell carcinoma, and large cell carcinoma. In addition, several rare subtypes exist, including neuroendocrine tumors, adenosquamous carcinoma, sarcomatoid carcinoma, salivary gland–type carcinomas, and SMARCA4-deficient undifferentiated tumors (SMARCA4-UT). Each histologic subtype of lung cancer possesses distinct cellular origins, morphologic characteristics, clinical behaviors, prognoses, epidemiologic features, risk factors, biomarker profiles, and responses to systemic therapies, including immunotherapy [4].

Immunotherapy has become a central pillar of modern cancer treatment [5]. By activating the patient’s immune system, it restores immune surveillance and enables sustained elimination of tumor cells. Immunotherapy can also remodel and enhance systemic immune function, thereby helping prevent tumor recurrence and metastasis. Among immunotherapeutic approaches, immune checkpoint inhibitors (ICIs) have revolutionized cancer management [6, 7], the diagnosis and therapeutic landscape across over 20 cancer types, including nearly all lung cancer histology and disease settings. In NSCLC lacking actionable mutations, ICIs are now established as standard therapy in several contexts: as neoadjuvant, perioperative, or adjuvant treatment for localized stage IB–III disease; as consolidation therapy following chemoradiation for stage II–III disease; and as monotherapy or in combination with platinum-based chemotherapy for advanced stages [4, 8]. In SCLC, ICIs are key components of therapy, used as consolidation following chemoradiation in limited-stage disease and in combination with platinum-based chemotherapy for extensive-stage disease [9, 10]. ICIs have demonstrated significant improvements in overall survival, quality of life, and the potential for durable responses or even cure in selected patients. Nevertheless, a substantial proportion of patients either fail to respond initially or develop acquired resistance, and second-line treatment options remain largely limited to chemotherapy [11]. Resistance to ICIs is complex and multifactorial. The primary mechanisms include insufficient tumor antigen recognition, impaired T-cell activation, tumor-driven immune evasion and exclusion, and the presence of physical or immunosuppressive barriers that restrict effective immune cell infiltration into the tumor microenvironment (TME) [12]. Moreover, effective ICI responses in NSCLC rely heavily on pre-existing cytotoxic CD8⁺ T-cell infiltration within tumor tissue [13].

The multifaceted nature of lung cancer and its TME necessitates innovative therapeutic strategies that target not only tumor cells but also the intricate interactions among stromal and immune components. While chimeric antigen receptor T (CAR-T) cells [14, 15], bispecific T-cell engagers (BiTEs) [16], and tumor-infiltrating lymphocytes (TILs) [17, 18] are promising immunotherapies for solid tumors as reviewed elsewhere [19], these approaches face significant challenges. These include high costs— often exceeding $1 million per patient—and limited efficacy in solid tumors due to the prevalent immunosuppressive TME [12, 20–22]. Cancer vaccines have emerged as a promising strategy to overcome these barriers. By inducing de novo or amplifying existing tumor-specific immune responses, cancer vaccines can enhance long-term immunological memory while minimizing the risk of autoimmunity. These features make them well-suited as immune stimulants for use alongside ICIs to improve therapeutic efficacy. This review aims to summarize the current evidence on cancer vaccine development in lung cancer, explore ongoing clinical efforts, and discuss future perspectives on how these vaccines can be integrated into the evolving landscape of immuno-oncology.

## History of cancer vaccines

### Milestones in cancer vaccination

The concept of cancer immunotherapy dates back to 1863, when Dr. Rudolf Virchow proposed the “chronic irritation theory,” linking chronic inflammation to tumor development. Although not a direct immunotherapy, this observation laid the foundation for understanding the immune system’s role in cancer. In the 1890 s, Dr. William B. Coley treated inoperable sarcomas with a mixture of heat-killed *Streptococcus pyogenes* and *Serratia marcescens* (Coley’s toxins), achieving notable clinical successes [23]. In the 1970 s, the intratumoral use of nonvirulent Mycobacterium bovis (Bacillus Calmette-Guérin, BCG) was shown to induce immune responses against various cancers, including cutaneous melanoma, partly through trained immunity [24]. In the post-genomic era, the first FDA-approved autologous dendritic cell (DC) vaccine, Sipuleucel-T, emerged in 2010 for metastatic castrate-resistant prostate cancer. Patient DCs are incubated with a fusion protein of prostatic acid phosphatase and GM-CSF, then reinfused to stimulate anti-tumor immunity. Talimogene laherparepvec (T-VEC), the first FDA-approved oncolytic virus in 2015, acts as an in-situ cancer vaccine in melanoma. Intratumoral injection lyses cancer cells, releasing tumor-associated and tumor-specific antigens (TAAs and TSAs) that, together with the virus, activate the immune system.

Virally induced cancers account for 15–20% of human malignancies, including HPV-related cervical and oropharyngeal cancers, HBV/HCV-related hepatocellular carcinoma, and EBV-associated lymphomas. Preventive vaccines against HPV and HBV have successfully reduced the incidence of these cancers, but such vaccines are not effective against established tumors, especially non-virus-related cancers like lung cancer [25]. Therapeutic vaccines targeting TAAs or TSAs from nonviral cancers have also shown limited efficacy when used alone.

**Fig. 1:**
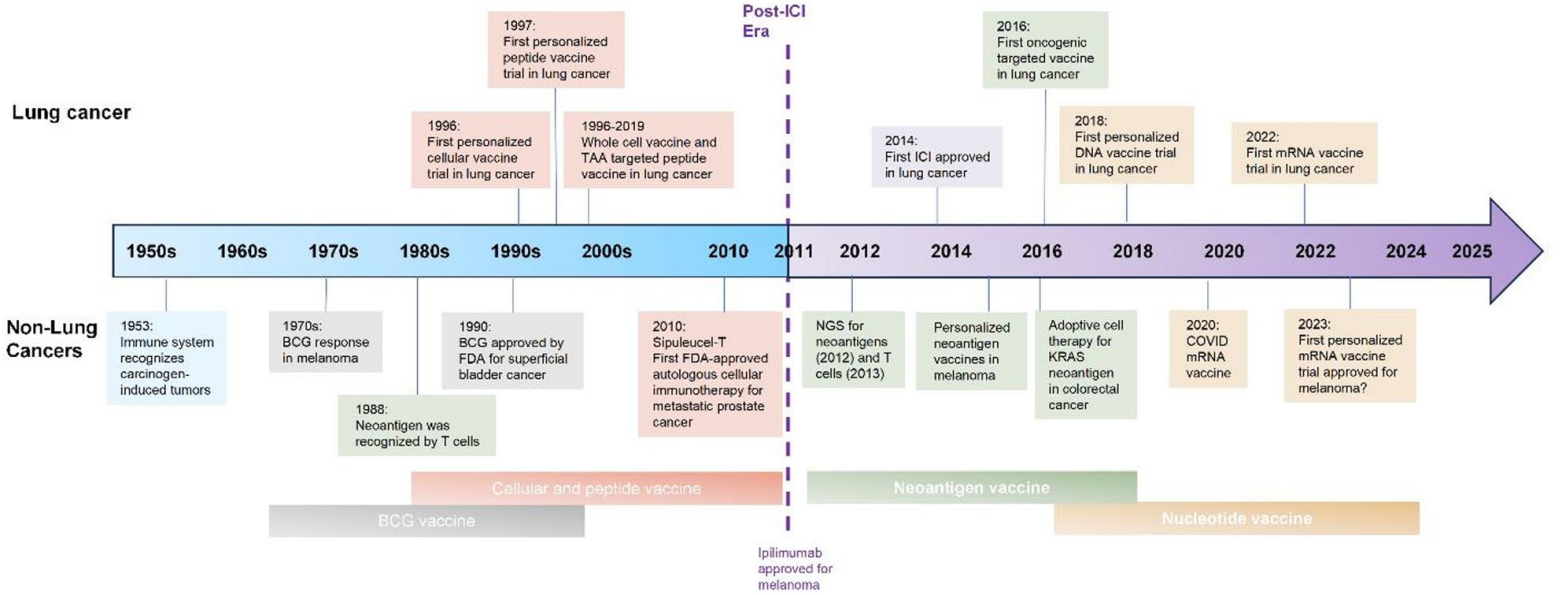
Milestones in Cancer Vaccine and ICI Development in Lung Cancer. Cancer vaccine strategies have evolved substantially alongside the development of immune checkpoint inhibitors (ICIs). Before the introduction of ICIs, vaccines were primarily developed as off-the-shelf formulations, such as peptide-based or dendritic cell-based vaccines, targeting tumor-associated antigens (TAAs), tumor-specific antigens (TSAs), or whole-cell tumor preparations. Since the first FDA approval of an ICI in 2011, vaccine research has increasingly emphasized combination approaches designed to overcome tumor-induced immunosuppression. More recently, the success of mRNA vaccines during the COVID-19 pandemic has accelerated interest in nucleic acid–based cancer vaccines, offering advantages in rapid manufacturing, scalability, and potent immunogenicity. BCG, Bacillus Calmette-Guérin; COVID-19, coronavirus disease 2019; FDA, U.S. Food and Drug Administration; ICI, immune checkpoint inhibitor; NGS, next-generation sequencing; TAA, tumor-associated antigen

Before ICIs, cancer vaccines were primarily off-the-shelf, including peptide-, DC-, or whole-cell-based vaccines (Table 1). Following FDA approval of ipilimumab in 2011, vaccines were increasingly combined with ICIs to overcome tumor-induced immunosuppression. This synergy allows vaccines to prime de novo T-cell responses, subsequently amplified by checkpoint blockade. Advances in next-generation sequencing (NGS) now enable personalized neoantigen vaccines, and the success of mRNA vaccines during the COVID-19 pandemic has accelerated nucleic acid–based cancer vaccine platforms, offering rapid production and strong immunogenicity (Fig. 1).

**Table 1:** Whole tumor cell-based lung cancer vaccine trials

Cancer type	NCT number	Disease setting	Trial status	Study phase	No. patient	Study period	Cellular base details	Combination	Vaccine name	Primary endpoint	Safety	Outcomes
NSCLC	NCT01829373	Stage I-IIIA (adjuvant)	Completed	II	N/A	2011-10 to 2013-02	Irradiated allogeneic tumor cells	GM-CSF	Vaccine 1650-G	Safety and immune response	N/A	N/A
NSCLC	NCT00654030	Stage I/II	Completed	II	12	2006-10 to 2009-11				Immune response	SAEs in 33.3% (suicidal attempt, pneumonia, dyspnea and arthroplasty)	6/11 (54.5% had immune response (measured by increased in interferon γ)
NSCLC	NCT00089726	Stage IIIB-IV	Completed	II	18	2003-03 to 2006-01	Irradiated allogeneic tumor cells	Chemotherapy	CG8123 (GVAX)	Tumor response	Most commonly grade 2 injection site reaction	ORR 0%, mPFS 5 mos, mOS 10 mos
NSCLC	NCT00074295	Stage IIIB-IV	Terminated	II	N/A	2004-03 to 2007-08		N/A		Safety, immune response, OS, and PFS	N/A	N/A
NSCLC	NCT00601796	Stage IV	Completed	II	19	2006-10 to 2012-06	Irradiated allogeneic tumor cells	Tretinoin and chemotherapy	N/A	Immune response	Headache (54%) and site reaction (38%)	ORR 0%, mPFS 1.7 mos, mOS 7.9 mos
NSCLC	NCT02466568	Stage IV	Withdrawn	I/II	N/A	2018-07 to 2020-07	Allogeneic tumor cells	ICI	N/A	Dose and ORR	N/A	N/A
NSCLC	NCT00503568	Stage IIIB-IV	Completed	I	19	2007-05 to 2012-08	Allogeneic tumor cells	N/A	gp96-vaccine	Safety	Injection site reaction. 4/19 (21%) SAEs unrelated to vaccine	ORR 32%, mOS 18 mos
NSCLC	NCT00676507	Stage IIIB-IV	Completed	III	270	2008-07 to 2013-01	Allogeneic tumor cells	N/A	Tergenpumatucel-L (Hyper-Acute(R)-Lung) (HS-110 Vaccine)	OS	No SAE	No difference in mOS (20.3 vs. 17.8 mos) in vaccine versus placebo
NSCLC	NCT02439450	Stage IV	Completed	I/II	121	2015-04 to 2022-11		Chemotherapy and ICI		Safety	SAEs: 0–27.66% across study arms	PD in 18/47, 41/68, 2/2, and 3/4 across study arms
Non-EGFR mutated NSCLC	NCT01504542	Locally advanced or metastatic	Withdrawn	II	N/A	2011-12 to 2013-12		Erlotinib		Immune response	N/A	N/A
NSCLC	NCT02117024	Advanced	Terminated	II	N/A	2014-07 to 2018-04		Chemotherapy		OS	N/A	N/A
NSCLC	NCT01774578	Stage IIIB-IV	Terminated	II/III	N/A	2013-02 to 2016-06		Chemotherapy		OS	N/A	N/A

*AE, adverse event; EGFR, epidermal growth factor receptor; GM-CSF, granulocyte-macrophage colony-stimulating factor; ICI, immune checkpoint inhibitor; mos, months; mOS, median overall survival; mPFS, median progression free survival; NSCLC, non-small cell lung cancer; ORR, objective response rate; OS, overall survival; PD, progressive disease; PFS, progression-free survival; SAE, severe adverse effect

### Mechanisms of therapeutic cancer vaccines in the era of ICIs

Lessons from the COVID-19 pandemic demonstrate that most lung cancer patients can mount adequate innate and adaptive immune responses to vaccines [26]. Vaccinology, the science of developing vaccines, has increasingly been applied to cancer treatment [27]. Therapeutic cancer vaccines are designed to elicit *de novo* T-cell responses by targeting one of two major types of cancer antigens [28, 29]: (1) **Tumor-Associated Antigens (TAAs)**: These are expressed at higher levels on cancer cells compared to normal cells and are relatively restricted to tumor cells; and (2) **Tumor-Specific Antigens (TSAs) or Cancer Neoantigens**: These result from genetic alterations that accumulate exclusively in cancer cells but not in normal cells during tumorigenesis or through epigenetic processes. Next-generation sequencing (NGS) and computational bioinformatics enable rapid identification of patient-specific neoantigens and assessment of immune responses. However, only ~10% of somatic mutations produce immunogenic peptides [30, 31]. To elicit an effective response, neoantigens must: [1] alter protein expression [2], be properly processed and presented on human leukocyte antigen (HLA) or human major histocompatibility complex (MHC) molecules for T-cell receptors (TCRs) recognition, and [3] exist as clonal or trunk rather than subclonal mutations. Compared with TAAs, neoantigens can bypass central tolerance and elicit stronger tumor immunity [28, 32].

**Fig. 2:**
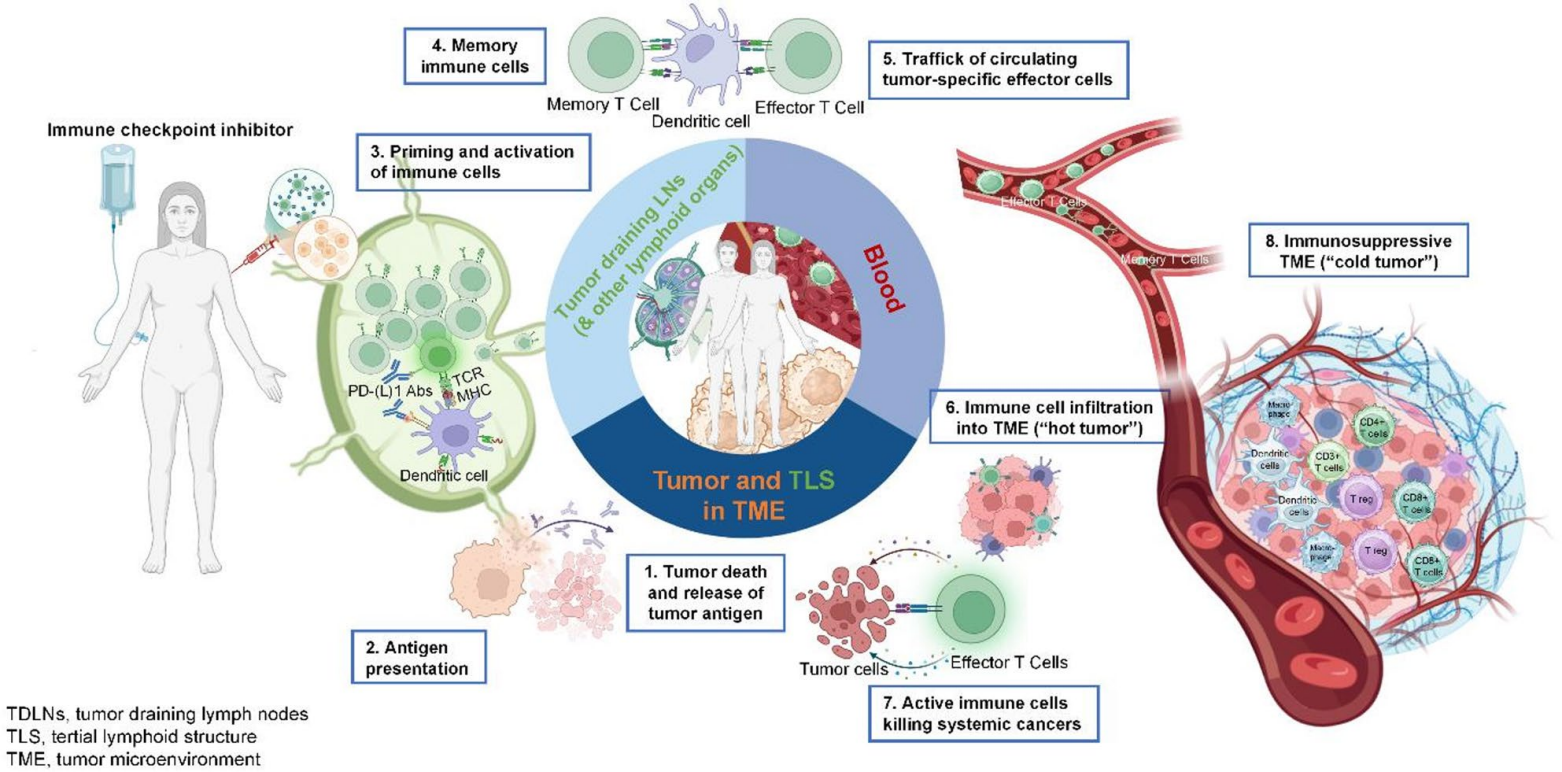
Mechanisms of Therapeutic Cancer Vaccines and Their Interaction with ICIs. Therapeutic cancer vaccines begin with the selection of tumor-associated or tumor-specific epitopes formulated with adjuvants to enhance immunogenicity. After administration, dendritic cells (DCs) capture and process the antigens, presenting them on HLA-I and HLA-II molecules in lymphoid tissues to activate CD8⁺ cytotoxic and CD4⁺ helper T cells. Effector CD8⁺ T cells infiltrate the tumor microenvironment (TME) to directly kill tumor cells, while CD4⁺ T cells promote B cell maturation, antibody production, and NK cell–mediated cytotoxicity. Booster doses strengthen memory T and B cell responses, supporting durable immune surveillance. By enhancing T cell activation and infiltration, vaccines—particularly when combined with immune checkpoint inhibitors (ICIs), can convert immunologically “cold” tumors into “hot” tumors, restoring effective antitumor immunity. Abs, antibodies; LN, lymph node; MHC, major histocompatibility complex; PD-L1, programmed death-ligand 1; TCR, T-cell receptor; TLS, tertiary lymphoid structure; TME, tumor microenvironment. Created with BioRender.com

Cancer vaccines train the immune system to recognize and attack tumor cells (Fig. 2). Antigenic epitopes, either public, shared, or personalized, are selected, combined with adjuvants, and administered via defined routes. Antigens are phagocytosed by dendritic cells (DCs), which present them on HLA-I and HLA-II molecules to prime CD8 + cytotoxic and CD4 + helper T cells in lymphoid tissues. CD4 + T cells facilitate maturation of CD8 + T cells and B cells, while B cells produce antibodies that mediate antibody-dependent cellular cytotoxicity (ADCC) via NK cells, releasing additional tumor antigens. Booster vaccinations amplify memory T and B cells, sustaining immune responses. This coordinated activation enhances anti-tumor immunity, which can be further potentiated by ICIs.

#### Types of vaccines

**Fig. 3:**
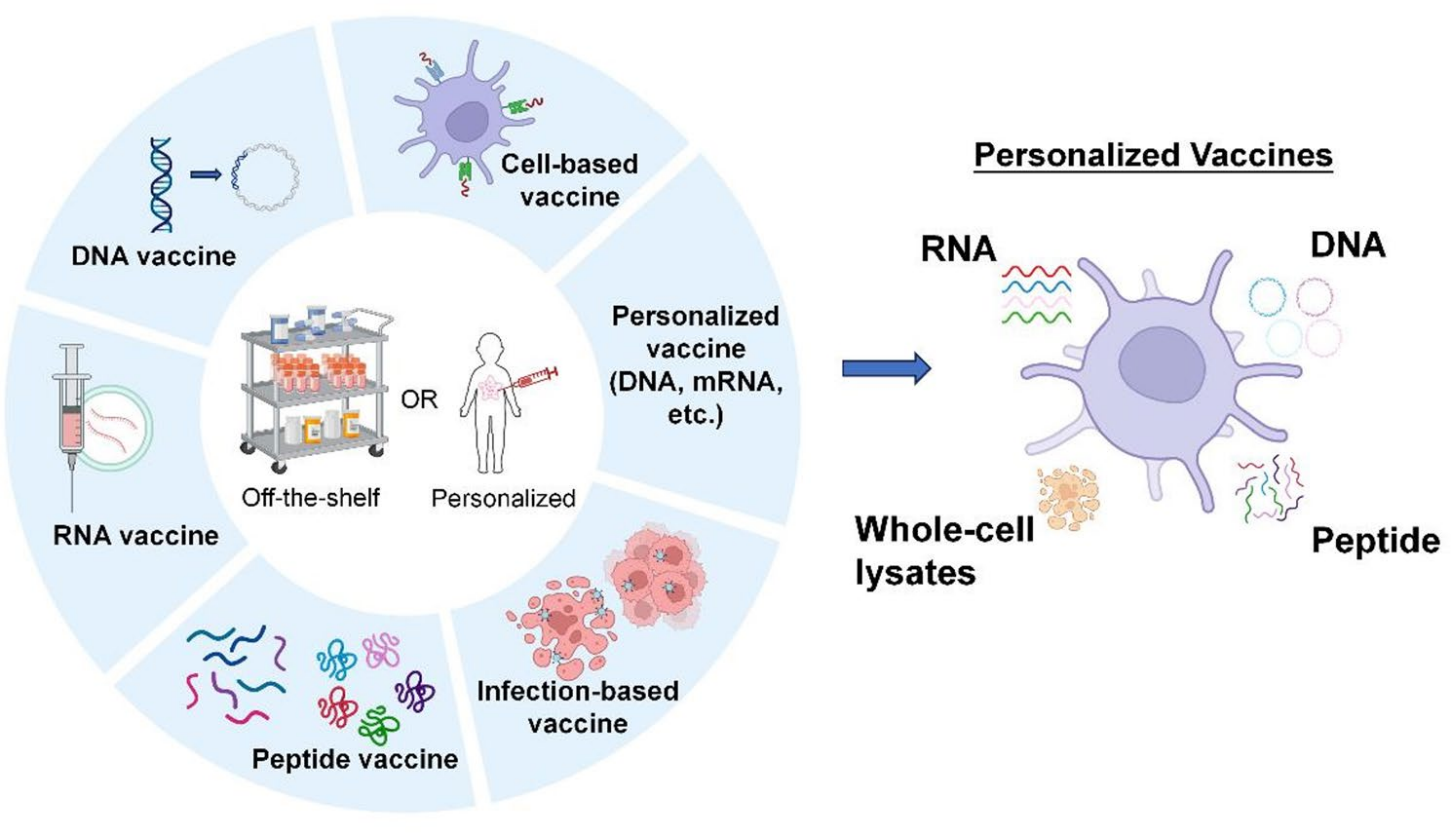
Types of Cancer Vaccine. Current cancer vaccine platforms include DNA, RNA, peptide, cell-based, and viral vector approaches. From a design perspective, they can be classified as either “off-the-shelf,” using shared targets, or “personalized,” designed around patient-specific targets. Created with BioRender.com

Selecting an appropriate delivery platform for cancer vaccines is a critical determinant of their success. Current vaccine platforms are categorized as DNA, RNA, peptide, cell-based, viral vector-based vaccines, and personalized mRNA vaccines (Fig. 3) (Table 2) [33, 34]. Tumor cell-based vaccines can be derived from autologous or allogeneic tumor cells [35], and may also be used in the form of tumor lysates [36]. Their advantages include the ability to present multiple antigens simultaneously and to induce immune responses that are not HLA-restricted. Subunit peptide vaccines are the most commonly used strategies [37]. These vaccines are either synthetically produced or purified from pathogens. However, their main limitations include low immunogenicity and a short duration of immune response [37]. DNA vaccines consist of plasmids encoding TAAs and other immunomodulatory molecules designed to enhance immune activation [38]. Transfected DNA plasmids enable stable expression and sustained antigen production; however, there remains a potential risk of the transfected DNA integration into the host genome. mRNA vaccines have recently emerged as a promising approach. They are synthesized via in vitro transcription using a bacteriophage RNA polymerase and a DNA template encoding the target antigen(s). Once internalized by host cells, they express proteins in the cytoplasm to elicit immune responses. mRNA vaccines are cost-effective and avoid the risk of genomic integration; however, they suffer from instability and inefficient in vivo delivery. Genetically modified oncolytic viral vaccines are engineered to selectively replicate within and eradicate tumor cells [39]. These viruses promote the release of autologous tumor antigens and can be further modified to deliver mRNA/DNA constructs or virus-like particles (VLPs) displaying neoantigen peptides [40]. One major advantage of viral vector-based vaccines is their strong immunogenicity. Delivering recombinant proteins through viral vectors elicits more potent immune responses than administering proteins with adjuvants [35]. Table 3 summarizes current clinical trials investigating viral vector–based vaccines in NSCLC.

**Table 2:** Types of cancer vaccines

Vaccine types	Advantages	Disadvantages
DNA vaccine	- Cost-effective and scalable production - No need for cell-based production - Capable of inducing sustained immune responses - Suitable for encoding multiple neoantigens and epitope types - Natural activation of immune responses via TLR9	- Potential genomic integration - Autoimmune risk - Low transfection efficiency
RNA vaccine	- Rapid design and manufacturing adaptability - No need for cell-based production - Suitable for encoding multiple neoantigens and epitope types - Strong immunogenicity and efficient uptake by APCs - Built-in immune stimulation via TLR3/7/8 - Short-lived expression limits long-term toxicity	- Short antigen expression window and rapid degradation - Can provoke systemic inflammation - Variable TLR7 response among patients
Peptide vaccine	- High antigen specificity and less off-target effects - No need for cell-based production - Compatible with direct MHC loading - Biodegradable and formulation-flexible	- Cost-intensive production - Less immunogenic alone and requires potent adjuvants - MHC allele-restricted presentation
Cell-based vaccine	- Potent activation of both innate and adaptive immunity - Capable of multiple neoantigen loading	- High cost - Need for patient-specific processing, cell isolation, and storage
Viral and bacterial vector vaccine (Infection-based vaccine)	- Naturally immunogenicity and built-in adjuvant effect from vectors - Enhanced memory T cells production	- Reduced booster effectiveness due to neutralizing immunity against vectors - Cost-intensive production - Complex storage and handling requirements
Personalized cancer vaccine	- Tailored to individuals - Durable immune response - Less immune evasion	- Time-consuming and expensive production - Challenges in predicting optimal neoepitopes and HLA-binding - Limited number of mutations can be included per formulation

TLR, Toll-like receptor; APC, Antigen-presenting cell; MHC, Major histocompatibility complex; HLA, Human leukocyte antigen

**Table 3:** Viral vector-based lung cancer vaccine trials

Cancer type	NCT number	Disease setting	Trial status	Study phase	No. patient	Study period	Combination	Vaccine name	Primary endpoint	Safety	Outcomes	Sponsor
NSCLC	NCT02140996	Stage IV	Unknown	I	21	2014-09 to 2017-06	N/A	Ad-sig-hMUC-1/ecdCD40L vector vaccine	Safety and dose	Grade 2 AEs: injection site reactions (71%), fever (10%), fatigue (5%), rash (5%)	ORR 0%, SD 48%, PD 33%	Singapore Clinical Research Institute
NSCLC	NCT00091039	Stage III after chemoradiation	Completed	Unknown	N/A	2004-08 to 2006-02	GM-CSF	Recombinant fowlpox-CEA(6D)/TRICOM vaccine	Safety	N/A	N/A	National Cancer Institute
NSCLC	NCT02879760	Unknown	Completed	I/II	N/A	2017-03 to 2020-05	ICI	Ad/MAGEA3 and MG1-MAGEA3	Safety, MTD and ORR	N/A	N/A	Turnstone Biologics, Corp.
SCLC	NCT00049218	Extensive stage	Completed	I/II	N/A	2003-04 to 2014-05	Chemotherapy	Ad.p53-DC	Safety	N/A	N/A	H. Lee Moffitt Cancer Center
SCLC	NCT00617409	Extensive stage	Completed	II	69	2007-10 to 2019-01	Chemotherapy and all-trans retinoic acid		Tumor response rate	Mostly grade 1 or grade 2 toxicities	No survival differences (ORR: 15.4%, 16.7%, and 23.8% for observatio, vaccine arm, and vaccine plus all-trans retinoic acid arm)	H. Lee Moffitt Cancer Center
SCLC	NCT03406715	Limited and extensive stage	Terminated	II	14	2018-03 to 2022-05	ICI		DCR	SAEs 100%, dyspnea 21.43%, confusion 14.29%, neoplasm 42.86%	mOS 120 days, mPFS 63 days, DCR 42.85%, ORR 21.4%	H. Lee Moffitt Cancer Center
Colorectal cancer, NSCLC, SCLC	NCT00088933	Stage IV	Terminated	I	N/A	2004-06 to Unknown	GM-CSF and chemotherapy	fowlpox-CEA-TRICOM	Safety, immune response and tumor response	N/A	N/A	NCI
Solid tumors	NCT01147965	Stage IV	Completed	I/II	32 colorectal cancer	2010-06 to 2013-03	N/A	Ad5[E1-, E2b-]-CEA Vaccine	Safety	Mostly mild injection site reaction (> 90%)	12-month OS 48%	Etubics Corporation
Solid tumors	NCT02179515	Locally advanced or metastatic	Completed	I	N/A	2014-06 to 2018-02	N/A	MVA-brachyury- TRICOM	Safety and MTD	Most grade 1–2 AEs. One grade 3 diarrhea	N/A	NCI
Solid tumors	NCT03639714	Stage IV	Completed	I/II	15	2019-02 to 2022-11	ICI	GRT-C901/GRT-R902	Safety, dose, and ORR	AEs > 10% included pyrexia, fatigue, musculoskeletal and injection site pain and diarrhea	Long-lasting neoantigen-specific CD8 T cell responses observed. SD in 4 out of 14 patients. One had CR	Gritstone bio, Inc.
Solid tumors	NCT02432963	Advanced stage	Ongoing	I	N/A	2016-06 to 2024-12	ICI	p53MVA vaccine	Safety	N/A	N/A	City of Hope Medical Center
Solid tumors	NCT03953235	Stage IV	Completed	I/II	19	2019-07 to 2023-03	ICI	GRT-C903/GRT-R904	Safety and ORR	Majority were grade 1/2 acute inflammation expected with viral vector and ICI	ORR 0%, mPFS 1.9 mo, and mOS 7.9 mo	Gritstone bio, Inc.
NSCLC, esophageal cancer	NCT04908111	Stage III-IV	Suspended	I/II	N/A	2021-12 to 2027-12	Chemotherapy and ICI	ChAdOx1-MAGEA3-NYESO, MVA-MAGEA3 and MVA-NYESO	Safety	N/A	N/A	Cancer Research UK
Melanoma and NSCLC	NCT04990479	Stage III/IV melanoma or NSCLC	Ongoing	I	N/A	2021-06 to 2024-03	ICI	NOUS-PEV	Safety	N/A	N/A	Nouscom SRL
CEA + solid tumors	NCT00529984	Stage IV	Completed	I/II	28	2007-09 to 2010-05	N/A	AVX701	Safety	2 grade 1 injection site reaction. 6 grade 3 events all attributed to disease progression.	Immune response observed (measured as CEA specific T cell response)	AlphaVax, Inc.
Solid tumors	NCT03384316	Refractory	Completed	I	10	2018-01 to 2020-08	None	ETBX-051, ETBX-061, and ETBX-011	Safety and dose	All TRAE were grade 1 or 2	Antigen specific T cell response observed	National Cancer Institute

*Ad5, adenovirus serotype 5; Ad.p53-DC, p53-transfected dendritic cell-based vaccine; AE, adverse event; AVX, alphavirus replicon particle; CEA, carcinoembryonic antigen; chemoRT, chemoradiation; CR, complete response; DC, dendritic cell; DCR, disease control rate; GM-CSF, granulocyte-macrophage colony-stimulating factor; ICI, immune checkpoint inhibitor; MAGE, melanoma-associated antigen; mOS, median overall survival; mPFS, median progression free survival; MTD, maximum tolerated dose; NSCLC, non-small cell lung cancer; ORR, objective response rate; OS, overall survival; PD, progressive disease; PFS, progression-free survival; SCLC, small cell lung cancer; SD, stable disease; TRAE, treatment-related adverse event; TRICOM, three costimulatory molecules (B7-1, ICAM-1, LFA)

## Vaccine targets in lung cancer

### Known non-oncogenic gene targets (traditional targets)

Carcinogens such as tobacco smoke, airborne particulate matter smaller than 2.5 microns (PM2.5), and chronic lung inflammation can induce somatic mutations in the bronchi, bronchioles, or alveoli. *Gain-of-function* mutations in oncogenes, including epidermal growth factor receptor (EGFR), Kirsten rat sarcoma viral oncogene homolog (KRAS), anaplastic lymphoma kinase (ALK), and human epidermal growth factor receptor 2 (HER2, also known as ERBB2), and loss-of-function mutations in tumor suppressor genes such as tumor protein p53 (TP53), RB transcriptional corepressor 1 (RB1), phosphatase and tensin homolog (PTEN), and cyclin-dependent kinase inhibitor 2 A (CDKN2A) drive malignant transformation. These driver mutations can accumulate over years or decades in histologically normal lung tissue or precancerous lesions, preceding the development of lung adenocarcinoma (LUAD) from atypical adenomatous hyperplasia (AAH), adenocarcinoma in situ (AIS), or minimally invasive adenocarcinoma (MIA), alongside numerous passenger mutations.

Mutations that occur in antigenic epitopes can generate neoantigens that are recognized as intolerable by the immune system. All nucleated cells, including cancer cells, express class I human leukocyte antigen (HLA-I) molecules, which present intracellular peptides (HLAp) on the cell surface. While epitopes from normally expressed proteins are tolerated by the immune system due to thymic selection, ectopically expressed proteins, such as cancer-testis antigens like New York esophageal squamous cell carcinoma 1 (NY-ESO-1), or neoantigens presented by HLA-I are recognized as non-self. Consequently, adaptive T cells mount immune responses against cancer cells. Innate immune natural killer (NK) cells also contribute by inducing apoptosis in stressed cancer cells [41].

**Fig. 4:**
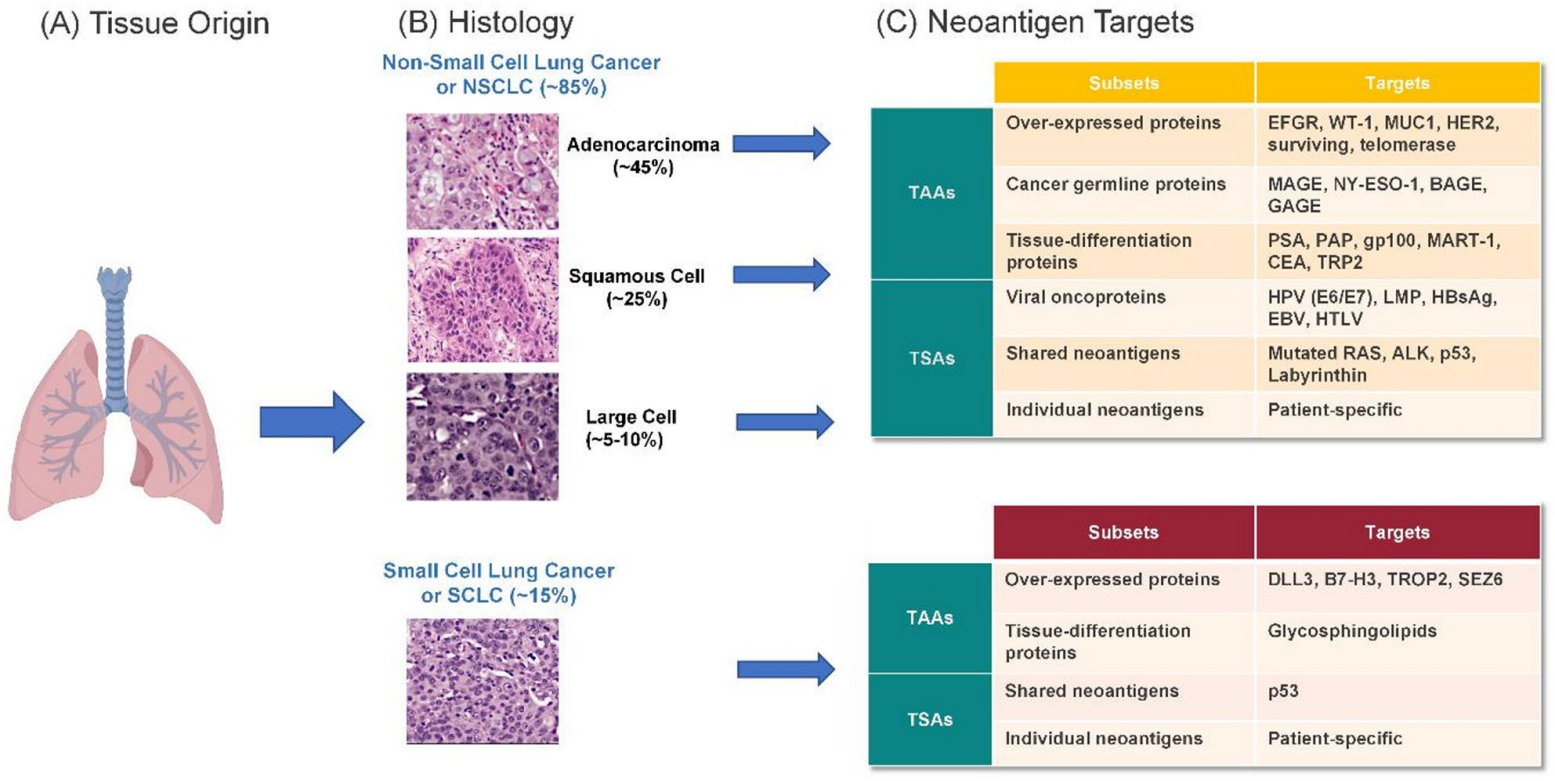
Neoantigens Targets in Lung Cancer. This figure summarizes key neoantigen targets in lung cancer, organized by histological subtype and further classified into tumor-associated antigens (TAAs) and tumor-specific antigens (TSAs). TAAs are proteins that are overexpressed in cancer cells relative to normal tissues and include overexpressed, cancer, germline, and tissue-differentiation antigens. TSAs, by contrast, result from genetic or epigenetic alterations unique to cancer cells and encompass viral oncoproteins as well as shared and patient-specific neoantigens. CEA, carcinoembryonic antigen; DLL3, delta-like canonical Notch ligand 3; EBV, Epstein–Barr virus; EGFR, epidermal growth factor receptor; HER2, human epidermal growth factor receptor 2; HBsAg, hepatitis B surface antigen; HPV, human papillomavirus; HTLV, human T-cell leukemia virus; LMP, latent membrane protein; MAGE, melanoma-associated antigen; MUC1, mucin 1; NSCLC, non-small cell lung cancer; PSA, prostate-specific antigen; RAS, rat sarcoma viral oncogene homolog; SCLC, small cell lung cancer; TAA, tumor-associated antigen; TSA, tumor-specific antigen; TRP2, tyrosinase-related protein 2; WT1, Wilms tumor gene 1. Created with BioRender.com

Professional antigen-presenting dendritic cells (DCs) phagocytose dying cancer cells and present TAAs and TSAs via HLA-I and HLA-II pathways. Naïve T cells primed by neoantigenic HLAp on DCs—especially those migrating from tumors to tumor-draining lymph nodes (TDLNs)—undergo clonal expansion and differentiate into effector CD8 + cytotoxic T lymphocytes (CTLs) and memory T cells. After entering circulation, these cells infiltrate tumors, where CTLs recognize HLAp-presenting cancer cells through TCRs, form immune synapses, and release perforins and granzymes to induce apoptosis. The immune response exerts selective pressure on the tumor, which may counteract via immune evasion mechanisms such as HLA-I downregulation, impaired antigen presentation, PD-L1 expression, and recruitment of immunosuppressive cells including regulatory T cells (Tregs), tumor-associated fibroblasts (TAFs), tumor-associated macrophages (TAMs), myeloid-derived suppressor cells (MDSCs), and tolerogenic DCs [42]. When immune responses dominate, tumors regress through epitope spreading; when tumors evade immunity, they progress, metastasize, and may ultimately lead to patient death. Previously investigated TAAs have yielded moderate clinical outcomes, which include mucin 1 (MUC1), melanoma-associated antigen (MAGE), NY-ESO, and carcinoembryonic antigen (CEA) (Tables 4 and 5; Fig. 4). Emerging targets such as HER2, Labyrinthin, T-cell Epitopes Associated with Impaired Peptide Processing (TEIPP), Wilms tumor gene 1 (WT1), and telomerase are now under active investigation.

**Table 4:** Ongoing lung cancer vaccines for single targets

Vaccine target	Cancer type	Vaccine type	NCT number	Disease setting	Trial status	Study phase	No. patients	Study period	Combination	Vaccine name	Primary endpoint	Safety	Outcomes	Sponsor
**CEA**	CEA + solid tumors	Yeast based	NCT00924092	Stage IV	Completed	I	25	2009-03 to 2012-08	None	GI-6207	Safety	Mostly grade 1/2 AEs	4/22 patients had SD	National Cancer Institute
	CEA + solid tumors	Virus-like replicon particle based	NCT00529984	Stage IV	Completed	I/II	28	2007-09 to 2010-05	None	AVX701	Safety	2 grade 1 injection site reaction; 6 grade 3 events	CEA-specific T cell response	AlphaVax, Inc.
	CEA + solid tumors	Personalized DC vaccine	NCT00128622	Stage IV	Completed	I	15	2005-09 to 2009-05	Denileukin Diftitox	CEA-TRICOM vaccine, GM-CSF	Safety	Rare grade 3 AEs	1/9 patients had minor response; 1 SD; 7 PD	H. Kim Lyerly
	CEA + solid tumors	Peptide vaccine	NCT00003125	Stage IV	Completed	II	N/A	1998-01 to 2004-11	IL-2 and GM-CSF	ALVAC-CEA and Vaccinia-CEA	Safety, immune response	N/A	N/A	Georgetown University
	Colorectal cancer, NSCLC, SCLC	Viral vector	NCT00088933	Stage IV	Terminated	I	N/A	2004-06 to N/A	GM-CSF and chemotherapy	fowlpox-CEA-TRICOM	Safety, immune response, tumor response	N/A	N/A	NCI
	NSCLC	Peptide vaccine	NCT00960115	Unresectable stage III	Completed	I/II	172	2008-12 to 2015-06	Chemotherapy	Tecemotide (L-BLP25)	OS	21.05% had SAEs, radiation pneumonitis (1.75%)	mOS 32.4 vs. 32.2 mos in vaccine vs. placebo arm	Merck KGaA, Darmstadt, Germany
	NSCLC	Viral vector	NCT00091039	Unresectable Stage III	Completed	Unknown	N/A	2004-08 to 2006-02	GM-CSF and chemoradiation therapy	Recombinant fowlpox-CEA(6D)/TRICOM vaccine	Safety	N/A	N/A	Philip M. Arlen, NCI
	CEA + solid tumors	Viral vector	NCT01147965	Stage IV	Completed	I/II	32 colorectal cancer	2010-06 to 2013-03	None	Ad5[E1-,E2b-]-CEA Vaccine	Safety	mild injection site reaction (> 90%)	12-month OS: 48%	Etubics Corporation
**HER2**	HER2 + solid tumors	Peptide vaccine	NCT00017537	Stage IV	Withdrawn	I	N/A	2000-03 to 2005-03	None	MVF-HER-2 (628–647)-CRL 1005	Optimum dose	N/A	N/A	University of Alabama at Birmingham
	HER2 + solid tumors	Peptide vaccine	NCT00005023	Stage III-IV	Completed	I	N/A	1999-03 to 2001-01	GM-CSF	HER-2/​Neu	Safety, immune response	N/A	N/A	University of Washington
	HER2 + solid tumors	Peptide vaccine	NCT00003002	Stage III-IV	Completed	I	N/A	1996-04 to 2004-01	GM-CSF	HER-2/​Neu	Safety, immune response	N/A	N/A	University of Washington
**NY-ESO-1**	NSCLC	Peptide vaccine	NCT02495636	Stage IIIB-IV	Withdrawn	II	N/A	2015-07 to 2017-07	ICI and poly-ICLC	CDX-1401	ORR	N/A	N/A	Yale University
	NY-ESO-1 + solid tumors	Antibody-peptide fusion vaccine	NCT01522820	High risk of recurrence or with minimal residual disease	Completed	I	N/A	2012-03 to 2016-07	Sirolimus	DC205-NY-ESO-1 vaccine (DEC-205/NY-ESO-1 fusion protein CDX-1401)	Safety	N/A	N/A	Roswell Park Cancer Institute
	NY-ESO-1 + solid tumors	Antibody-peptide fusion vaccine	NCT00948961	Progressed on standard therapy	Completed	I/II	45	2009-09 to 2014-02	Resiquimod and poly-ICLC	CDX-1401	Safety	No DLTs or grade 3 toxicities	16/45 patients had SD; 2 had PR	Celldex Therapeutics
	Lung, ovarian cancer and melanoma	Peptide vaccine	NCT01584115	Unknown	Unknown	I/II	N/A	2012-07 to 2013-07	Adjuvant Monophosphoryl Lipid A (MPLA)	N/A	Safety	N/A	N/A	Instituto de Investigação em Imunologia
	NY-ESO-1 or LAGE-1 + solid tumors	DNA vaccine	NCT00199849	Failed standard therapy	Completed	I	18	2004-09 to 2007-09	None	pPJV7611	Safety	No DLTs	No clinical response	Ludwig Institute for Cancer Research
**MAGE-A3**	NSCLC	Viral vector	NCT02879760	Unknown	Completed	I/II	N/A	2017-03 to 2020-05	ICI	Ad/MAGEA3 and MG1-MAGEA3	Safety, MTD and ORR	N/A	N/A	Turnstone Biologics, Corp.
	NSCLC	Peptide vaccine	NCT00290355	Stage IB-II	Completed	II	182	2002-05 to 2011-07	None	GSK 249,553	Disease recurrence	14% and 13% patients had grade 3 toxicities	No difference in DFS or OS	GlaxoSmithKline
**MAGE 12**	MAGE-12 + solid tumors	Peptide vaccine	NCT00020267	Stage IV	Completed	I	N/A	2000-07 to N/A	IL-2 and Montanide ISA-51	N/A	Safety and immune response	N/A	N/A	NCI
**MUC1**	NSCLC	Peptide vaccine	NCT02823990	Recurrent disease	Completed	II	N/A	2016-12 to 2021-02	ICI	TG4010 (modified vaccinia virus Ankara MVA- MUC1-IL2 vaccine)	ORR	N/A	N/A	Karen Kelly, University of California, Davis
	NSCLC	Peptide vaccine	NCT03353675	Stage IIIB-IV	Completed	II	44	2018-01 to 2021-02	IL-2, chemotherapy and ICI		ORR	SAEs: 63.6% pts, general health deterioration (20.45%), DVT (4.55%)	ORR 32.5%; mPFS 5.7 mos; mOS 14.9 mos	Transgene
	NSCLC	Peptide vaccine	NCT00415818	Stage IIIB-IV	Completed	II/III	148	2005-12 to 2010-03	IL2		PFS	Fever (23.3%), abdominal pain (16.4%), injection site pain (5.5%)	6-mo PFS: 43.2% vs. 35.1% in vaccine- chemotherapy versus chemotherapy	Transgene
	NSCLC	Peptide vaccine	NCT00828009	Unresectable stage IIIA-IIIB	Completed	II	N/A	2011-01 to 2019-05	Bevacizumab	Tecemotide (BLP25)	Safety	N/A	N/A	ECOG-ACRIN Cancer Research Group
	NSCLC	Peptide vaccine	NCT00157196	Unresectable stage IIIA	Terminated	N/A	N/A	2012-01 to 2015-08	Chemotherapy		Safety	N/A	N/A	Merck KGaA
	NSCLC	Peptide vaccine	NCT00157209	Stage IIIB, IV	Completed	II	N/A	2000-08 to 2012-07	Chemotherapy		Safety	N/A	N/A	Merck KGaA
	NSCLC	Peptide vaccine	NCT00409188	Unresectable stage III	Completed	III	1513	2007-01 to 2015-04	Chemotherapy		OS	Grade 3/4 AEs: dyspnea (5%), CNS metastasis (3%), pneumonia (2%)	No OS difference (25.6 vs. 22.3 mos)	EMD Serono
	NSCLC	Viral vector	NCT02140996	Stage IV	Unknown	I	21	2014-09 to 2017-06	None	Ad-sig-hMUC-1/ecdCD40L vector vaccine	Safety and dose	Grade 2 AEs: injection site reactions (71%), fever (10%), fatigue and rash (5%)	ORR 0%, SD 48%, PD 33%	Singapore Clinical Research Institute
	NSCLC	Peptide vaccine	NCT03300817	Prevention	Ongoing	I	N/A	2017-12 to 2025-12	Poly-ICLC	N/A	Safety, immune response	N/A	N/A	NCI
	NSCLC	RNA vaccine	NCT03164772	Stage IV	Completed	I/II	57	2017-12 to 2021-10	ICI	BI1361849	Safety	SAEs: 4.3% vs. 8.8% in vaccine + single ICI vs. vaccine + dual ICI	PFS: 2 vs. 1.8 mos	Ludwig Institute for Cancer Research
	NSCLC and neuroendocrine carcinoid tumors	Peptide vaccine	NCT01720836	Stage I-III	Ongoing	I/II	N/A	2012-11 to 2029-09	Poly-ICLC	N/A	Immune response	N/A	N/A	Olivera Finn
**Telomerase**	Solid tumors	DNA vaccine	NCT00753415	Stage I-III NSCLC	Completed	I	37	2008-08 to 2011-04	None	V934/V935	Safety	No SAEs or DLT	Antigen- specific immune response	Merck Sharp & Dohme LLC
	NSCLC	Peptide vaccine	NCT01579188	Unresectable stage III	Unknown	III	N/A	2012-05 to 2016-05	None	GV1001	OS	N/A	N/A	Kael-GemVax Co., Ltd.
	NSCLC	Peptide vaccine	NCT01935154	Stage IV or recurrent stage I-III	Completed	II	221	2012-08 to 2017-01	None	Vx001	OS	No > grade 2 treatment toxicity	No difference in mOS (11.3 vs. 14.3 mos); ORR 0%	Vaxon Biotech
	NSCLC	Peptide vaccine	NCT04263051	Refractory advanced stage	Ongoing	II	N/A	2020-09 to 2025-09	ICI	UCPVax	PFS	N/A	N/A	Centre Hospitalier Universitaire de Besancon
	NSCLC	Peptide vaccine	NCT02818426	Stage IV	Ongoing	I/II	N/A	2016-04 to 2023-12	None	UCPVAx	DLT and immune response	N/A	N/A	Centre Hospitalier Universitaire de Besancon
	NSCLC	Peptide vaccine	NCT01789099	Stage III-IV	Completed	I/II	18	2013-04 to 2023-04	GM-CSF	UV1	Safety and immune response	No SAEs	15/17 pts SD, 2 PD; mPFS 10.7 mos; mOS 28.2 mos	Ultimovacs ASA
	NSCLC	Peptide vaccine	NCT05344209	Inoperable stage IIIB/IIIC or stage IV	Ongoing	II	N/A	2022-08 to 2027-07	ICI	UV1	PFS	N/A	N/A	Vestre Viken Hospital Trust
**Glycosphingolipids**	Solid tumors	Glycosphingolipids vaccine	NCT01349647	After standard therapy	Completed	I	N/A	2011-05 to 2015-07	KLH and OPT-821	N/A	Safety, immune response	N/A	N/A	Memorial Sloan Kettering Cancer Center
	SCLC	Anti-idiotype vaccine and bacterial based vaccine	NCT00003279; NCT00037713	Limited stage SCLC	Completed	III	N/A	1998-03 to N/A	N/A	Bec2/Bacille Calmette-Guerin	Safety, clinical efficacy	N/A	N/A	European Organisation for Research and Treatment of Cancer
**Survivin**	Prostate, ovarian, and NSCLC	Peptide vaccine	NCT05104515	Locally advanced or metastatic	Ongoing	I	N/A	2021-11 to 2024-12	None	OVM-200	Safety	N/A	N/A	Oxford Vacmedix UK Ltd.
	Neuroendocrine tumor of gastrointestinal, pancreatic or lung origin	Peptide vaccine	NCT06202066	Stage IV	Ongoing	II	N/A	2024-10 to 2028-10	Chemotherapy, Freund’s adjuvant, GM-CSF, and octreotide acetate	SVN53-67/M57-KLH peptide vaccine (SurVaxM)	PFS	N/A	N/A	Roswell Park Cancer Institute
	Neuroendocrine tumor of gastrointestinal, pancreatic or lung origin	Peptide vaccine	NCT03879694	Stage IV	Ongoing	I	N/A	2019-06 to 2025-12	Freund’s adjuvant, GM-CSF, and octreotide acetate		Safety	N/A	N/A	Roswell Park Cancer Institute
**gp96-Ig**	NSCLC	Whole cell vaccine	NCT01799161	Stage IIIB, IV or relapsed	Withdrawn	I	N/A	2014-12 to N/A	Oxygen and Theophylline	gp96-Ig Vaccine	Safety	N/A	N/A	Eckhard Podack
**Polysialic acid**	SCLC	Carbohydrate based vaccine	NCT00004249	Any stages after standard therapy	Completed	II	18	1998-08 to 2001-11	QS-21	NP-polySA–KLH	Safety and dose	Grade 3 ataxia of unclear etiology: 1/18 patients	increased antigen specific antibodies	Memorial Sloan Kettering Cancer Center
**WT1**	WT + solid tumors, AML	Peptide vaccine	NCT03761914	Stage IV	Ongoing	I/II	N/A	2019-06 to 2023-04	ICI	Galinpepimut-S	Safety and clinical efficacy	N/A	N/A	Sellas Life Sciences Group
	Solid and hematological malignancy	Peptide vaccine	NCT02498665	Advanced Stage	Completed	I	24	2015-11 to 2018-09	None	DSP-7888	Safety and dose	No DLT, grade 1/2 injection site reaction	4/24 SD, 16 PD, 4 not evaluable, mOS 180 days.	Sumitomo Pharma America, Inc.
	WT + NSCLC, mesothelioma, AML, MDS	Peptide vaccine	NCT00398138	Stage III-IV	Completed	I	N/A	2006-10 to 2009-06	GM-CSF, Freund’s adjuvant	N/A	Safety, immune response	N/A	N/A	Memorial Sloan Kettering Cancer Center
**TEIPP**	NSCLC	Peptide vaccine	NCT05898763	Relapsed advanced stage	Completed	I	26	2021-09 to 2024-07	ICI and Montanide	LRPAP7-30 V-SLP	Safety, immune response	23 SAEs but mostly ascribed to disease rather than intervention	1 PR, 8 SD, and 2 mixed responses	Erasmus Medical Center
**uGcGM3 tumor-associated ganglioside**	NSCLC	Anti-idiotype vaccine	NCT01240447	Stage IIIA, IIIB, and IV	Completed	II	N/A	2009-09 to 2014-06	Chemotherapy	Racotumomab	Safety, immune response	N/A	N/A	Laboratorio Elea Phoenix S.A.
	NSCLC	Anti-idiotype vaccine	NCT01460472	Unresectable stage III-IV	Unknown	III	N/A	2010-09 to 2016-09	Chemotherapy	Racotumomab	Safety, PFS	N/A	N/A	Recombio SL
**Arginase-1**	Solid tumor	Peptide vaccine	NCT03689192	Metastatic disease	Completed	I	10	2018-12 to 2022-01	Montanide	N/A	Safety	No vaccine-related grade 3–4 AEs	Antigen specific immune response	Herlev Hospital
**CLAUDIN-6**	CLDN6-positive tumor	RNA vaccine	NCT04503278	Metastatic or unresectable disease	Ongoing	I	N/A	2020-09 to 2040-01	CART	CLDN6 RNA-LPX	Safety	N/A	N/A	BioNTech Cell & Gene Therapies GmbH
**PRAME and PSMA**	Solid tumor	DNA + peptide vaccine	NCT00423254	Advanced refractory disease	Completed	I	N/A	2007-02 to 2009-11	None	MKC1106-PP	Safety, immune response	N/A	N/A	Mannkind Corporation
**URLC10**	NSCLC	Peptide vaccine	NCT01069640	Refractory to Standard Therapy	Completed	I	N/A	2010-02 to 2019-03	Montanide	N/A	Safety	N/A	N/A	Shiga University
	NSCLC	Peptide vaccine	NCT01949701	Advanced stage	Completed	I/II	N/A	2011-08 to 2019-03	Montanide	N/A	Safety, clinical efficacy	N/A	N/A	Shiga University
**p53**	Solid tumors	Viral vector	NCT02432963	Advanced	Ongoing	I	11	2016-06 to 2024-12	ICI	p53MVA vaccine	Safety	1 grade 5 myocarditis	3 SD	City of Hope Medical Center
	SCLC	DC vaccine	NCT00776295	Limited stage SCLC	Terminated	II	N/A	2007-05 to 2010-08	Chemotherapy, aPBSCT	N/A	OS	N/A	N/A	H. Lee Moffitt Cancer Center and Research Institute
**Labyrinthin**	Pan-adenocarcinoma	Peptide vaccine	NCT05101356	Advanced and metastatic adenocarcinomas	Phase I completed; Phase II ongoing	I/II	N/A	2021-10 to 2030-01	ICI and GM-CSF	Labvax 3(22)−23	ORR, Safety, PFS, OS in Phase II	N/A	N/A	Tianhong Li, University of California, Davis

*AML, acute myeloid leukemia; AE, adverse event; aPBSCT, autologous peripheral blood hematopoietic cell transplantation; CEA, carcinoembryonic antigen; CLDN6, claudin-6; DC, dendritic cell; DFS, disease free survival; DLT, dose limiting toxicity; DVT, deep vein thrombosis; GM-CSF, granulocyte-macrophage colony-stimulating factor; HER2, human epidermal growth factor receptor 2; ICI, immune checkpoint inhibitor; IL-2, interleukin-2; KLH, keyhole limpet hemocyanin; MDS, myelodysplastic syndromes; mOS, median overall survival; mPFS, median progression free survival; MTD, maximum tolerated dose; MUC1, mucin-1; NSCLC, non-small cell lung cancer; ORR, objective response rate; OS, overall survival; PD, progressive disease; PFS, progression-free survival; PSMA, prostate-specific membrane antigen; PR, partial response; PRAME, preferentially expressed antigen in melanoma; RNA, ribonucleic acid; SCLC, small cell lung cancer; SD, stable disease; TAA, tumor-associated antigen; TEIPP, T-cell epitopes associated with impaired peptide processing; TSA, tumor-specific antigen; WT1, Wilms tumor gene 1

**Table 5:** Ongoing trials of multi-target vaccines for lung cancer

Vaccine target	Cancer type	Vaccine type	NCT number	Disease setting	Trial status	Study phase	No. patients	Study period	Combination	Vaccine name	Primary endpoint	Safety	Outcomes	Sponsor
**CEA**,**4 p53**,**5**,**6 HER-2/neu**,**7**,**8**,** MAGE 2 and 39**	NSCLC	Peptide vaccine	NCT00104780	Unresectable IIIB or IV	Unknown	II	N/A	2004-12 to N/A	None	EP2101	OS and safety	N/A	N/A	Epimmune
**B7.1**,** ICAM-1**,** and LFA-**	Solid tumors	Viral vector	NCT02179515	Unresectable IIIB or IV	Completed	I	38	2014-06 to 2018-02	None	MVA-brachyury- TRICOM	Safety and MTD	Most grade 1–2 AEs; 1 grade 3 diarrhea	N/A	National Cancer Institute (NCI)
**CEA**,** MUC1**,** brachyury**	Solid tumors	Viral vector	NCT03384316	Refractory	Completed	I	10	2018-01 to 2020-08	None	ETBX-051, ETBX-061, and ETBX-011	Safety and dose	All TRAEs were grade 1 or 2	Antigen specific T cell response observed	NCI
**NY-ESO-1**,** MAGE-A3/A4**,** Multi-MAGE-A**,** MUC1**,** Survivin**,** Melan-A**	NSCLC	DC vaccine	NCT03970746	Stage II-IV	Ongoing	I/II	N/A	2019-09 to 2025-12	ICI and chemotherapy	PDC*lung01	Safety	N/A	N/A	PDC*line Pharma SAS
**NY-ESO-1**,** MAGEC1/2**,** 5 T4**,** survivin**,** MUC1**	NSCLC	RNA vaccine	NCT01915524	Stage IV	Terminated	I	N/A	2013-04 to 2016-07	Radiation therapy	CV9202	Safety	N/A	N/A	CureVac
**KOC1**,** TTK**,** CO16**,** DEPDC1**,** MPHOSPH1**	Cervical, GI, and Lung	Peptide vaccine	NCT00676949	Stage IV	Completed	I	N/A	2007-11 to 2010-03	Chemotherapy	N/A	Safety	N/A	N/A	Kyushu University
**Three cancer-testis antigens**	NSCLC	Peptide vaccine	NCT01592617	Advanced stage	Unknown	II	N/A	2012-05 to 2015-09	None	S-488,410	Safety, immune response	N/A	N/A	Shiga University
**URLC10**,** CDCA1**,** VEGFR1**,** VEGFR2**	NSCLC	Peptide vaccine	NCT00874588	Advanced or recurrent disease	Completed	I	N/A	2009-03 to 2012-06	Montanide	N/A	Safety	N/A	N/A	Fukushima Medical University
**URLC10**,** CDCA1**,** and KIF20A**	NSCLC	Peptide vaccine	NCT01950156	Advanced stage in remission	Completed	I/II	N/A	2011-09 to 2019-03	Montanide	N/A	Safety, efficacy	N/A	N/A	Shiga University
**URLC10**,** VEGFR1 and VEGFR2 + TTK or CDCA1**	NSCLC	Peptide vaccine	NCT00633724	Advanced or recurrent disease	Completed	I	N/A	2007-05 to 2012-06	Montanide	N/A	Safety	N/A	N/A	Fukushima Medical University
**DEPDC1**,** MPHOSPH1**,** URLC10**,** CDCA1 and KOC1**	Solid tumor	Peptide vaccine	NCT04316689	Unresectable, metastatic, or recurrent disease	Completed	I	7	2019-07 to 2021-09	None	S-588,210	Safety	No DLT, 2 Grade3 AEs (hypertension, injection site reaction)	Antigen specific T cell response observed	Shionogi
**URLC10**,** TTK and KOC1**	NSCLC	Peptide vaccine	NCT00674258	Advanced or recurrent disease	Unknown	I/II	N/A	2008-05 to 2009-04	Montanide	N/A	Safety	N/A	N/A	Tokyo University
**URLC10**,** CDCA1**,** and KIF20A**	NSCLC	Peptide vaccine	NCT01069575	Advanced or failed standard therapy	Completed	I	N/A	2010-02 to 2019-03	Montanide	N/A	Safety	N/A	N/A	Shiga University
**CDCA1 and KIF20A**	SCLC	Peptide vaccine	NCT01069653	Refractory to standard therapy	Completed	I	N/A	2010-02 to 2019-03	Montanide	N/A	Safety	N/A	N/A	Shiga University
**NYESO-1**,** melanoma antigen family C1/C2**,** survivin**,** trophoblast**	NSCLC	RNA vaccine	NCT00923312	Stage IIIB-IV	Completed	I/II	46	2009-05 to 2014-05	None	CV9201	Safety and dose	Most AEs were grade 1/2. 3 grade 3 AEs (7%)	mPFS 5.6 mos, mOS 10.8 mos. 31% SD; 69% PD	CureVac
**MAGE-A3 and NY-ESO-1**	NSCLC, esophageal cancer	Viral vector	NCT04908111	Stage III-IV	Suspended	I/II	N/A	2021-12 to 2027-12	Chemotherapy and ICI	ChAdOx1-MAGEA3-NYESO, MVA-MAGEA3, MVA-NYESO	Safety	N/A	N/A	Cancer Research UK
**HER-2/neu**,** CEA**,** MAGE 2**,** MAGE 3 and p53**	NSCLC	Peptide vaccine	NCT02654587	Metastatic disease failed ICI	Terminated	III	N/A	2016-02 to 2021-01	None	OSE2101	OS	N/A	N/A	OSE Immunotherapeutics
**HER-2/neu**,** CEA**,** MAGE 2/3**,** p53**	NSCLC	Peptide vaccine	NCT06472245	Metastatic disease failed ICI	Ongoing	III	N/A	2024-06 to 2028-12	None	OSE2101	OS	N/A	N/A	OSE Immunotherapeutics
**CEA**,** p53**,** HER-2/neu**,** MAGE 2/3**	NSCLC	Peptide vaccine	NCT00054899	Stage IIb-IIIa	Completed	I/II	63	2003-01 to N/A	None	EP-2101	Safety, immune response	N/A	Antigen specific T cell response observed	Epimmune
**CDH3**,** CD105**,** YB-1**,** MDM2**,** SOX2**	NSCLC	DNA vaccine	NCT05242965	Stage IV	Ongoing	II	N/A	2023-03 to 2026-12	GM-CSF	STEMVAC	Safety, immune response	N/A	N/A	University of Washington
**Multiple TAAs**	Primary or metastatic lung cancer	mRNA vaccine	NCT06928922	Advanced stage	Ongoing	I	N/A	2025-02 to 2028-02	ICI	BMD006	Safety	N/A	N/A	Cancer Institute and Hospital, Chinese Academy of Medical Sciences
**Multiple TAAs**	NSCLC	RNA vaccine	NCT05557591	Stage IIIB, IIIC, or IV	Ongoing	II	N/A	2023-04 to 2027-06	ICI	BNT116	Safety and ORR	N/A	N/A	Regeneron
**Multiple TAAs**	NSCLC	RNA vaccine	NCT05142189	Unresectable Stage III or IV	Ongoing	I	N/A	2022-06 to 2027-08	Chemotherapy and ICI	BNT116	Safety	N/A	N/A	BioNTech

*AE, adverse event; CEA, carcinoembryonic antigen; CDCA1, cell division cycle-associated 1; DC, dendritic cell; DEPDC1, DEP domain containing 1; GM-CSF, granulocyte-macrophage colony-stimulating factor; ICAM-1, intercellular adhesion molecule 1; ICI, immune checkpoint inhibitor; KIF20A, kinesin family member 20 A; LFA, lymphocyte function-associated antigen; MAGE, melanoma-associated antigen; MDM2, mouse double minute 2 homolog; mOS, median overall survival; mPFS, median progression free survival; MTD, maximum tolerated dose; MUC1, mucin-1; mRNA, messenger RNA; NSCLC, non-small cell lung cancer; ORR, objective response rate; OS, overall survival; p53, tumor protein p53; PD, progressive disease; PR, partial response; RNA, ribonucleic acid; SCLC, small cell lung cancer; SD, stable disease; SOX2, SRY-box transcription factor 2; TAA, tumor-associated antigen; TRAE, treatment-related adverse event; TTK, threonine and tyrosine kinase; VEGFR, vascular endothelial growth factor receptor; WT1, Wilms tumor gene 1

### Emerging oncogenic targets in lung cancer

Targetable oncogenic mutations are identified in approximately 60% of lung adenocarcinoma patients in Western populations and 80% in Asian populations [43, 44]. Commonly identified oncogenes in NSCLC include EGFR, ALK rearrangements, KRAS, ROS1, BRAF V600E, NTRK1/2/3, MET exon 14 skipping mutations, RET, and ERBB2 (HER2). Targeted therapies using tyrosine kinase inhibitors (TKIs) have achieved substantial clinical success in these patient populations; however, therapeutic resistance typically develops within months to years of treatment [45]. Evidence indicates that patients harboring these oncogenic mutations generally exhibit minimal response to ICIs, likely due to low tumor mutation burden and reduced immunogenicity [46–49]. These observations highlight the need for novel therapeutic strategies in oncogene-driven NSCLC. The widespread adoption of NGS has facilitated the rapid identification of targetable oncogenes, positioning them as promising candidates for the development of “off-the-shelf” vaccine therapies.

#### KRAS

KRAS is the most frequently mutated oncogene in NSCLC, occurring in approximately 20–25% of cases [50], and represents the most extensively studied target for cancer vaccines. A combined vaccine regimen, consisting of chimpanzee adenovirus (ChAd68) and self-amplifying mRNA (samRNA), targeting multiple RAS and CTNNB mutations in combination with ICIs, is currently under investigation in metastatic solid tumors (NCT03953235) [51]. Among the 19 patients enrolled, 18 harbored KRAS mutations. Phase I interim results demonstrated that the vaccine was well tolerated, with 31% of patients exhibiting T-cell responses. However, no objective response rates (ORRs) were observed; 8 of 19 patients (42%) achieved stable disease (SD), while 11 patients experienced disease progression. In the 6 NSCLC patients enrolled, a mixed response was noted, with most progression occurring within the first two months, likely before a sufficient T-cell response could be mounted. These findings suggest that although an immune response can be induced, a clinically meaningful antitumor effect was not observed. Several ongoing KRAS-targeting vaccine trials employ diverse platforms, including peptide-based, nucleotide-based, and viral vector–based vaccines. Combination strategies are also being explored, incorporating immune stimulators, ICIs, and CAR-T cell therapies (Table 6). Of note, one ongoing Phase I trial is evaluating the safety and feasibility of administrating the KRAS peptide vaccine with poly-ICLC adjuvant in combination with nivolumab and ipilimumab for first-line treatment of patients with unresectable Stage III/IV NSCLC harboring specific KRAS mutations (NCT05254184, 2025 WCLC, P3.18.40).

**Table 6:** Cancer vaccine trials targeting oncogenic mutations

Cancer target	Cancer type	Vaccine type	NCT number	Disease setting	Trial status	Study phase	No. patients	Study period	Combination	Vaccine name	Primary endpoint	Safety	Outcomes
**EGFR**	NSCLC	Peptide vaccine	NCT04298606	Prevention and survivor (stage IA-IIIA)	Ongoing	I	N/A	2021-11 to 2024-11	None	CIMAvax Vaccine	Safety, immune response	N/A	N/A
	NSCLC, squamous head and neck cancer	Peptide vaccine	NCT02955290	IV	Ongoing	I/II	23	2016-12 to 2027-12	ICI	CIMAvax Vaccine	Safety, OS, PFS	N/A	DCR 47.6%. mOS 11.9 mos
	EGFR + NSCLC	Peptide vaccine	NCT06095934	Stage IIIB-IV (EGFR-TKI resistance)	Ongoing	I/II	N/A	2022-03 to 2025-12	ICI and chemotherapy	N/A	ORR	N/A	N/A
**ALK**	ALK + NSCLC	Peptide vaccine	NCT05950139	Stage IV	Ongoing	I/II	N/A	2024-05 to 2029-07	ALK inhibitors	N/A	Safety, immune response	N/A	N/A
**Multiple RAS and CTNNB**	Solid tumors (pancreatic cancer, NSCLC, CRC)	Viral vector and mRNA vaccine	NCT03953235	Stage IV	Completed	I/II	19	2019-07 to 2023-03	ICI	GRT-C903/GRT-R904	Safety, ORR	Majority grade 1/2 acute inflammation	ORR 0%, mPFS 1.9 mos, mOS 7.9 mos
**KRAS**	KRAS + (G12V or G12D) solid tumors	Peptide vaccine	NCT06253520	Stage IV	Ongoing	I	N/A	2024-05 to 2033-06	CART and IL-2	GRT-C903/GRT-R904	Safety, CR and/or PR	N/A	N/A
	KRAS/NRAS+ (G12D or G12R) solid tumors	Oligonucleotide and peptide vaccine	NCT04853017	Stage IV	Ongoing	I	N/A	2021-10 to 2026-03	none	ELI-002	MTD, safety	N/A	N/A
	KRAS + (G12C, G12V, G12D, G12A, G13D or G12R) NSCLC	Peptide vaccine	NCT05254184	Unresectable stage III and stage IV	Ongoing	I	N/A	2022-11 to 2027-04	poly-ICLC adjuvant and ICI	N/A	Safety	N/A	N/A
	KRAS + (G12A/C/D/R/S/V, or G13D) NSCLC and pancreatic cancer	Peptide vaccine	NCT06015724	Refractory	Ongoing	II	N/A	2024-01 to 2026-01	Daratumumab and ICI	Targovax TG-01/Stimulon QS-21	ORR	N/A	N/A
	KRAS + (G12D, G12V, G13D or G12C) solid tumors	mRNA vaccine	NCT03948763	Stage IV	Completed	I	N/A	2019-06 to 2022-08	ICI	mRNA-5671/V941	Safety	N/A	N/A
	KRAS + (codon 12) NSCLC	Peptide vaccine	NCT00005630	Stage IB-IV	Completed	I	N/A	1999-07 to 2002-05	GMCSF	N/A	Safety, immune response	N/A	N/A
**RAS**	RAS + solid tumors	Peptide vaccine	NCT00019331	Stage IV	Completed	II	N/A	1997-10 to 2007-05	GM-CSF, Detox-PC and IL-2	N/A	Safety, immune response	N/A	N/A
	Pancreatic, CRC, SCLC, and NSCLC	Peptide vaccine	NCT00019006	Stage III-IV	Completed	I	N/A	1995-03 to Unknown	Detox-B	N/A	MTD, safety	N/A	N/A

*ALK, anaplastic lymphoma kinase; CART, chimeric antigen receptor T cell; CRC, colorectal cancer; CR, complete response; CTNNB, catenin beta; DCR, disease control rate; EGFR, epidermal growth factor receptor; GM-CSF, granulocyte-macrophage colony-stimulating factor; ICI, immune checkpoint inhibitor; IL-2, interleukin-2; mOS, median overall survival; mPFS, median progression free survival; MTD, maximum tolerated dose; mRNA, messenger RNA; NSCLC, non-small cell lung cancer; ORR, objective response rate; OS, overall survival; PFS, progression-free survival; poly-ICLC, polyinosinic-polycytidylic acid stabilized with poly-L-lysine and carboxymethylcellulose; PR, partial response; RAS, rat sarcoma viral oncogene homolog; SCLC, small cell lung cancer; TKI, tyrosine kinase inhibitor

#### ALK

ALK inhibitors have been among the most successful target therapies in NSCLC. The most recent 5-year follow-up data from the CROWN study demonstrated a progression-free survival (PFS) of 60% in the lorlatinib group [52]. To prevent or delay the development of resistance to ALK inhibitors, an ALK peptide vaccine was developed and has shown immunogenicity in preclinical studies [53]. A phase I/II clinical trial of this vaccine (NCT05950139) is currently underway in patients with ALK-rearranged NSCLC receiving ALK inhibitors, with primary endpoints evaluating treatment-related adverse events and vaccine-specific immune responses.

#### EGFR

Similarly, vaccine therapy is also being developed in patients with EGFR mutations who progressed on tyrosine kinase inhibitors (TKIs). Ongoing studies are focused on neoantigens generated from tyrosine kinase mutations such as EGFR L858R and EGFR exon 19 deletions [54, 55]. A recent single-center study in China investigated a neoantigen vaccine combined with tislelizumab and chemotherapy in patients with stage IIIB–IV EGFR-mutated NSCLC who had progressed after EGFR-TKIs. With a median follow-up of 24 months, preliminary results demonstrated both immune and clinical responses. Among 11 patients who received the EGFR neoantigen vaccine, 45.5% achieved an ORR, and 100% achieved disease control (DCR)—including 5 patients with SD and 6 patients with partial response (PR) without severe adverse events. Immune monitoring in 7 patients revealed that 85.7% demonstrated vaccine-induced T-cell responses against EGFR neoantigen peptides (NCT06095934) [56].

Oncogene-targeted vaccine therapy has broader applications beyond the EGFR-mutated population. CIMAvax-EGF, a recombinant anti-human epidermal growth factor (EGF) vaccine, is being developed for NSCLC irrespective of EGFR mutation status. A Phase II trial of CIMAvax-EGF combined with nivolumab as second-line therapy showed survival benefits, with a 47.6% DCR and a 3-year OS rate of 29%. Notably, subgroup analysis revealed that only 8% of PD-L1–negative patients achieved PFS compared with 38% of PD-L1–positive patients (NCT02955290) [57]. Nevertheless, early-phase trials of CIMAvax-EGF in preventive and early-disease settings are ongoing, aiming to evaluate immune responses and safety in patients at high risk for lung cancer and in those with IB–IIIA early-stage NSCLC who have undergone curative treatment (NCT04298606).

### Personalized cancer vaccine

In contrast to off-the-shelf cancer vaccines, which target tumor antigens commonly shared among patients with the same cancer type, personalized cancer vaccines are designed to target patient-specific neoantigens [58]. While neoantigens are generated by tumor-specific genetic alterations, the highly polymorphic nature of HLA molecules determines which neoantigen-derived peptides can be effectively presented and recognized by T cells in each patient. As a result, the set of immunogenic neoantigens is largely patient-specific, providing the biological rationale for personalized vaccine strategies aimed at selectively targeting tumor-restricted mutations. This personalized approach is hypothesized to be more effective in targeting certain somatic cancer mutations. In NSCLC, tumor mutational burden (TMB) varies significantly by patient, smoking, oncogene mutation status, sample type (tissue or blood), inter- and intra-patient tumor heterogeneity, and sequencing and analytic platforms. Tumors with high TMB (≥ 10 mutations/Mb) generate a larger repertoire of neoantigens, thereby increasing the pool of candidate targets for personalized cancer vaccine development [59]. Tables 7 and 8 summarize completed and ongoing personalized vaccine trials.

**Table 7:** Completed, personalized lung cancer vaccine trials

Vaccine type	Cancer type	NCT number	Disease setting	Trial status	Study phase	No. patient	Study period	Combination	Vaccine name	Primary endpoint	Safety	Outcomes	Sponsor
**Personalized DC vaccine**	NSCLC	NCT00442754	Advanced	Completed	II	N/A	2006-12 to 2009-11	Celecoxib, imiquimod and IL-2	MelCancerVac	Immune response	N/A	N/A	Herlev Hospital
	NSCLC	NCT00103116	Stage IA-IIIB	Completed	II	16	2004-10 to 2008-04	N/A	N/A	Immune response, clinical response	No SAEs	6/16 patients antigen- specific immune response	Edward Hirschowitz
	NSCLC	NCT00023985	Adjuvant (stage IB-IIIA)	Completed	I	N/A	2001-01 to 2003-08	N/A	N/A	Safety, immune response	N/A	N/A	Roswell Park Cancer Institute
	NSCLC	NCT00601094	Stage IIIB-IV	Completed	I	N/A	2009-02 to 2017-05	N/A	Adenovirus CCL21 vaccine (Ad-CCL21-DC)	Safety, MTD	N/A	N/A	Jonsson Comprehensive Cancer Center
	NSCLC	NCT01574222	Stage IIIB-IV	Terminated	I	16	2011-10 to 2017-03	N/A		Safety, MTD	4 grade 1 TRAEs (flu-like symptoms, hemoptysis, nausea, and fatigue)	Antigen specific immune response. mOS 3.9 mos, SD 25%	VA Office of Research and Development
	NSCLC	NCT00098917	Adjuvant (stage IB-IIIA)	Terminated	I	N/A	2005-02 to Unknown	N/A	N/A	Safety, MTD	N/A	N/A	Jonsson Comprehensive Cancer Center
	NSCLC	NCT02956551	Refractory	Unknown	I	N/A	2016-11 to 2020-06	N/A	N/A	Safety	N/A	N/A	Sichuan University
	NSCLC	NCT04082182	Stage IV	Unknown	I	N/A	2019-08 to 2021-12	N/A	N/A	Safety, MTD	N/A	N/A	University Hospital, Ghent
	NSCLC	NCT01398124	Stage IA-IIIA (adjuvant)	Withdrawn	Unclear	N/A	2012-12 to 2015-12	N/A	N/A	Immune response	N/A	N/A	Milton S. Hershey Medical Center
	NSCLC and SCLC	NCT03871205	Refractory	Unknown	I	N/A	2019-04 to 2020-12	N/A	N/A	Safety, immune response	N/A	N/A	Shenzhen People’s Hospital
	p53 mutant NSCLC	NCT00019929	Adjuvant (stage IIIA/IIIB)	Completed	II	N/A	2000-08 to 2005-12	N/A	Mutant p53 peptide pulsed DC vaccine	Safety, immune response, OS	N/A	N/A	National Cancer Institute (NCI)
	SCLC	NCT00049218	Extensive stage	Completed	I/II	N/A	2003-04 to 2014-05	Chemotherapy	Ad.p53-DC	Safety	N/A	N/A	H. Lee Moffitt Cancer Center
	SCLC	NCT00617409	Extensive stage	Completed	II	69	2007-10 to 2019-01	Chemotherapy and all-trans retinoic acid (ATRA)	Ad.p53-DC	Tumor Response Rate	Mostly grade 1 or grade 2 toxicities	No OS differences (ORR: 15.4%, 16.7%, and 23.8% for observation, vaccine, and vaccine plus ATRA arm)	H. Lee Moffitt Cancer Center
	CEA + solid tumors	NCT00128622	Stage IV	Completed	I	15	2005-09 to 2009-05	denileukin diftitox	N/A	Safety	Rare grade 3 AEs attributed to disease progression	1/9 patients had minor response; 1 SD; 7 PD	H. Kim Lyerly
	SCLC	NCT03406715	Limited and extensive stage	Terminated	II	14	2018-03 to 2022-05	ICI	Ad.p53-DC	DCR	SAEs 100%, include dyspnea 21.43%, confusion 14.29%, neoplasm 42.86%	mOS 120 days, mPFS 63 days, DCR 42.85%, ORR 21.4%	H. Lee Moffitt Cancer Center
**Personalized cellular vaccine**	Lung, esophageal, thymic, thoracic sarcomas, pleural mesotheliomas	NCT01258868	Adjuvant	Terminated	I	N/A	2010-12 to 2016-06	Celecoxib and ISCOMATRIX	N/A	Safety	N/A	N/A	NCI
	NSCLC	NCT05642195	Stage IB-IIIA	Suspended	I/II	N/A	2024-10 to 2035-12	Montanide (R) ISA-51 VG and the IL-15 Super-Agonist N-803	H1299 Cell Lysates	Safety and immune response	N/A	N/A	NCI
	NSCLC	NCT00298298	Adjuvant (stage IA-IIIA)	Terminated	I/II	N/A	2006-01 to 2014-01	N/A	L-Vax	Safety and immune response	N/A	N/A	AVAX Technologies
	NSCLC	NCT00793208	Adjuvant after surgical resection	Terminated	I	N/A	2008-12 to 2015-11	N/A	N/A	Safety and feasibility	N/A	N/A	Theresa Whiteside, PhD
	Sarcomas, Melanomas, Germ Cell Tumors, or Epithelial Malignancies	NCT01341496	Stage IV	Terminated	I	N/A	2011-04 to 2016-07	Chemotherapy, celecoxib and ISCOMATRIX	N/A	Safety	N/A	N/A	NCI
	Solid tumors	NCT01061840	Advanced	Completed	I	74	2009-12 to 2019-01	N/A	Autologous Vigil™ vaccine	Safety	No grade 3 or higher AEs	mOS 562 months	Gradalis, Inc.
	Solid tumors	NCT00019084	Advanced	Completed	II	N/A	1996-02 to 2003-05	IL-2	N/A	Immune response	N/A	N/A	NCI
	Solid tumors	NCT00722228	Stage IV	Unknown	I/II	N/A	2008-07 to 2022-01	N/A	N/A	Unknown	N/A	N/A	Hadassah Medical Organization
**Tumor-derived autophagosome vaccine**	NSCLC	NCT00850785	Stage IIB-IV	Completed	I	4	2009-01 to 2012-05	Chemotherapy and GM-CSF	Dribbles	Immune response	Most grade 1/2 AEs; 3 grade 5 AEs (sepsis, pneumonia)	2/3 patients immune response; All 4 patients: PD	Providence Health & Services
	NSCLC	NCT03057340	Adjuvant (stage IIIA/IIIB)	Unknown	I	N/A	2017-06 to 2020-12	GM-CSF or miquimod		PFS	N/A	N/A	Second Affiliated Hospital, Zhejiang University
**Personalized neoantigen peptide vaccine**	NSCLC	NCT00098085	Adjuvant (stage IB-IIIA)	Completed	II	N/A	2003-09 to 2007-11	N/A	Heat-shock protein peptide complex-96 (HSPPC-96)	Feasibility	N/A	N/A	Agenus Inc.
	NSCLC	NCT03380871	Unresectable or metastatic disease	Completed	I	38	2018-05 to 2021-02	Chemotherapy, ICI, poly-ICLC	NEO-PV-01 vaccine	Safety	Only TRAE was injection site reaction (29%). No SAEs	ORR 69%. Immune response in 100% patients	BioNTech US Inc.
	NSCLC	NCT04998474	Advanced	Unknown	II	N/A	2022-01 to 2024-07	ICI	N/A	Immune response	N/A	N/A	Frame Pharmaceuticals B.V.
	NSCLC and mesothelioma	NCT00003974	Stage I-IIIA NSCLC, stage I-II mesothelioma	Completed	I	N/A	1997-08 to 2000-11	Chemotherapy, DetoxPC	N/A	Immune response	N/A	N/A	Roswell Park Cancer Institute
	NSCLC and SCLC	NCT03166254	Stage IV	Withdrawn	I	N/A	2019-04 to 2027-05	ICI, poly-ICLC	NEO-PV-01 vaccine	Safety and feasibility	N/A	N/A	Washington University
	NSCLC, melanoma, and bladder cancer	NCT02897765	Unresectable or metastatic disease	Completed	I	82	2016-10 to 2020-05	ICI, poly-ICLC	NEO-PV-01 vaccine	Safety	No serious TRAEs	ORR 39% in NSCLC, 59% in melanoma, 27% in bladder cancer	BioNTech US Inc.
	Solid tumors	NCT01065441	Stage II-IV	Completed	I/II	N/A	2010-12 to 2013-07	AlloStim	N/A	Safety	N/A	N/A	Michael Har-Noy
	Solid tumors	NCT00861107	Stage IV	Completed	I/II	N/A	2009-08 to 2011-05	AlloStim	N/A	Safety	N/A	N/A	Mirror Biologics, Inc.
	Solid tumors	NCT03633110	Stage IV	Completed	I/II	16	2018-08 to 2022-02	ICI	GEN-009 vaccination	Safety and immune response	No SAEs	Durable immune response observed	Genocea Biosciences, Inc.
	Solid tumors	NCT03715985	Unresectable or metastatic	Unknown	I/II	12	2019-01 to 2022-12	ICI	NeoPepVac	Safety	Only grade 1/2 TRAEs	ORR 67%	Herlev Hospital
**Personalized DNA vaccine**	Solid tumors	NCT03548467	Locally advanced or metastatic	Completed	I/II	N/A	2018-04 to 2023-01	bempegaldesleukin (NKTR-214)	VB10.NEO	Safety	N/A	N/A	Nykode Therapeutics ASA
**Personalized mRNA vaccine**	Solid tumors	NCT03639714	Stage IV	Completed	I/II	15	2019-02 to 2022-11	ICI	GRT-C901/GRT-R902	Safety, dose, and ORR	AEs > 10% included pyrexia, fatigue, musculoskeletal, injection site pain, diarrhea	Long-lasting antigenspecific CD8 + T cell responses. SD 4/14; 1 CR	Gritstone bio, Inc.

*Ad-CCL21-DC, adenovirus-CCL21-transfected dendritic cell vaccine; Ad.p53-DC, p53-transfected dendritic cell-based vaccine; AE, adverse event; AlloStim, allogeneic T cell stimulation therapy; CEA, carcinoembryonic antigen; CR, complete response; DC, dendritic cell; DCR, disease control rate; GM-CSF, granulocyte-macrophage colony-stimulating factor; HSPPC-96, heat shock protein peptide complex-96; ICI, immune checkpoint inhibitor; IL-2, interleukin-2; MTD, maximum tolerated dose; mOS, median overall survival; mPFS, median progression free survival; mRNA, messenger RNA; NSCLC, non-small cell lung cancer; ORR, objective response rate; ORD, Office of Research and Development; ORR, objective response rate; OS, overall survival; PFS, progression-free survival; SAE, serious adverse event; SCLC, small cell lung cancer; SD, stable disease; PD, progressive disease; poly-ICLC, polyinosinic-polycytidylic acid stabilized with poly-L-lysine and carboxymethylcellulose; TME, tumor microenvironment

**Table 8:** Ongoing personalized lung cancer vaccine trials

Vaccine type	Cancer type(s)	NCT number	Disease setting	Study phase	Study period	Combination	Vaccine name	Primary endpoint	Sponsor
**Personalized DC vaccine**	NSCLC	NCT06752057	Second-line treatment in advanced disease	II	2024-09 to 2027-02	ICI, radiotherapy	N/A	ORR	The First Affiliated Hospital of Nanchang University
	NSCLC	NCT06751849	Third-line treatment in advanced disease	II	2024-03 to 2026-06	ICI, radiotherapy	N/A	ORR	The First Affiliated Hospital of Nanchang University
	Gastric, hepatocellular, lung, and colorectal cancers	NCT04147078	Adjuvant (locally advanced disease)	I	2019-06 to 2026-06	N/A	N/A	DFS	Sichuan University
	NSCLC	NCT03546361	Stage IV	I	2019-07 to 2025-01	ICI	autologous DC-adenovirus CCL21 vaccine	MTD, ORR	Jonsson Comprehensive Cancer Center
	NSCLC	NCT05195619	Stage IIIA-IV	I	2021-12 to 2024-09	Chemotherapy	N/A	Safety	Centre Hospitalier Universitaire Vaudois
	NSCLC and SCLC	NCT05886439	Stage IIIB-IV or extensive stage	I/II	2023-05 to 2026-12	ICI	LK101	Safety	Cancer Institute and Hospital, Chinese Academy of Medical Sciences
	NSCLC	NCT06329908	Stage IIIB-IV	I	2023-09 to 2026-10	ICI	Neo-DCVac	Safety	Zhen-Yu Ding
	NSCLC	NCT04078269	Adjuvant (stage IA-IIIA)	I	2019-08 to 2025-12	N/A	N/A	Safety	University Hospital, Ghent
	SCLC	NCT04487756	Extensive stage	I/II	2021-03 to 2024-10	Chemotherapy and ICI	N/A	Safety and PFS	Instituto Oncológico Dr Rosell
**Personalized neoantigen vaccine (peptide)**	Solid tumors	NCT05269381	Advanced	I/II	2022-03 to 2026-02	ICI, GM-CSF, chemotherapy	PNeoVCA	Safety	Mayo Clinic
	NSCLC and head and neck cancer	NCT04266730	Refractory	I	2024-12 to 2033-06	ICI and Poly-ICLC	PANDA-VAC	Safety	UNC Lineberger Comprehensive Cancer Center
	NSCLC	NCT06751901	Third-line treatment in advanced disease	II	2024-03 to 2026-06	ICI, radiotherapy	N/A	ORR	The First Affiliated Hospital of Nanchang University
	NSCLC	NCT06752044	Second-line treatment in advanced disease	II	2024-09 to 2027-02	ICI, radiotherapy	N/A	ORR	The First Affiliated Hospital of Nanchang University
	NSCLC, breast cancer, and melanoma	NCT05098210	Stage III-IV	I	2022-06 to 2026-11	Poly-ICLC and ICI	N/A	Safety	Fred Hutchinson Cancer Center
**Personalized DNA vaccine**	SCLC	NCT04397003	Extensive stage	II	2022-03 to 2030-03	ICI	N/A	Safety and feasibility	Washington University School of Medicine
**Personalized mRNA vaccine**	NSCLC	NCT06735508	Resectable disease	I	2025-01 to 2026-12	ICI	N/A	Safety	Guangdong Provincial People’s Hospital
	NSCLC	NCT06685653	Stage IIB-IV	II	Unknown to 2026-11	ICI	RGL-270	Safety	Nanjing Tianyinshan Hospital
	Melanoma and NSCLC	NCT04990479	Unresectable stage III/IV melanoma or NSCLC	I	2021-06 to 2024-10	ICI	NOUS-PEV	Safety	Nouscom SRL
	NSCLC	NCT06077760	Resected stage II-IIIB	III	2023-12 to 2035-12	ICI	mRNA-4157 (V940)	DFS	Merck Sharp & Dohme LLC
	NSCLC	NCT06623422	Resectable Stage II-IIIB	III	2024-10 to 2033-05	ICI		DFS	Merck Sharp & Dohme LLC
	Solid tumors	NCT03313778	Adjuvant NSCLC	I	2017-08 to 2026-06	ICI		Safety	ModernaTX, Inc.
	Solid tumors	NCT03289962	Locally advanced or metastatic	I	2017-12 to 2025-03	ICI	autogene cevumeran (RO7198457)	Safety and dosage	Genentech, Inc.
	NSCLC and esophageal cancer	NCT03908671	Stage IIIB-IV	Unclear	2019-10 to 2025-12	N/A	N/A	Safety	Stemirna Therapeutics

*Ad-CCL21-DC, adenovirus-CCL21-transfected dendritic cell vaccine; DFS, disease-free survival; GM-CSF, granulocyte-macrophage colony-stimulating factor; ICI, immune checkpoint inhibitor; IL-2, interleukin-2; MTD, maximum tolerated dose; mRNA, messenger RNA; NSCLC, non-small cell lung cancer; ORR, objective response rate; PFS, progression-free survival; Poly-ICLC, polyinosinic-polycytidylic acid stabilized with poly-L-lysine and carboxymethylcellulose; SCLC, small cell lung cancer

Current delivery platforms for personalized vaccines mirror those of off-the-shelf approaches and include peptide-based, nucleic-based, cell-based, or engineered viral vector-based formats. However, the manufacturing process can be more complex. It involves biospecimen acquisition, NGS mutation profiling, neoantigen identification with immunogenicity prediction, vaccine synthesis, and final product release. The typical turnaround time from sample collection to vaccine administration is approximately 3–4 months, posing significant challenges in patients with rapidly progressing disease [33, 60].

#### Personalized DNA vaccine

A phase I/IIa study of VB10.NEO, a DNA plasmid vaccine, was conducted in 3 clinical study sites in Germany. VB10.NEO contains up to 20 neoepitopes selected by proprietary AI platform NeoSELECT and is designed to target antigen presenting cells via Nykode’ s modular Vaccibody™ platform. In the interim report released in 2023, 41 patients had received at least one dose of VB10.NEO. All patients demonstrated immune responses to a minimum of three neoepitopes, including neoepitope-specific polyfunctional CD8 + T cell responses. The breadth and magnitude of these immune responses were observed to be dose-dependent [61].

#### Personalized RNA vaccine

Following the success of mRNA-4157(V940), an individualized mRNA neoantigen cancer vaccine in resected melanoma [62], the same agent is now being studied in solid tumors, including NSCLC, in the phase I KEYNOTE-603 trial (NCT03313778). The study comprises two parts: Part A includes patients in adjuvant setting receiving mRNA-4157 as monotherapy post-surgery; Part B includes patients with metastatic disease receiving the vaccine in combination with pembrolizumab. Four NSCLC patients were included in Part A. The therapy was well tolerated, with mostly grade 1 and 2 expected toxicities, without any grade 4 or 5 adverse events. Immunogenicity was confirmed with both *de novo* T-cell responses and enhancement of pre-existing T-cell responses. Longitudinal follow up for 100 days after last dose of vaccine showed durable neoantigen specific T-cell responses [63]. Two additional phase III trials evaluating V940 in combination with pembrolizumab versus pembrolizumab alone are actively recruiting patients in the adjuvant settings (NCT06623422, NCT06077760).

Autogene cevumeran is another personalized mRNA vaccine currently being evaluated in a phase I trial for solid tumors, including NSCLC, both with and without atezolizumab (NCT03289962). In a non-prespecified interim analysis, three grade 4/5 treatment-related adverse events were reported: grade 4 pancreatitis, grade 4 systemic inflammatory response syndrome, and grade 5 pneumonitis. Notably, the vaccine induced poly-epitope neoantigen-specific T-cell responses involving both CD4 + and CD8 + cells, demonstrating both the strength and diversity of the immune response. These responses were detectable for up to 23 months after treatment initiation.

An individualized vaccine regimen combining self-amplifying mRNA (samRNA) and heterologous chimpanzee adenovirus (ChAd68) with dual ICIs, nivolumab and ipilimumab, is being assessed in patients with metastatic solid tumors in an ongoing phase I/II trial (GRANITE trial, NCT03639714). Phase I interim results demonstrated feasibility and induction of long-lasting T-cell responses [64]. A single priming dose of ChAd68 was administered at week 0, followed by multiple samRNA boosts at weeks 4, 8, 12, 16, 20, 24, 36, and 48 with escalating doses. Immunogenicity data were available for 13 of 14 patients, with neoantigen-specific CD8 + T-cell responses detected in 100% of participants. Notably, T-cell responses were durable, with levels of 700–5,000 SFU/10⁶ cells maintained for over 52 weeks in three patients. Only one NSCLC patient was enrolled; this patient exhibited a seven-fold increase in CD8 + T-cell response at weeks 24 and 36 post-boosts, though the duration of response remains to be determined. Serious treatment-related adverse events included one case each of pyrexia, duodenitis, elevated transaminases, and hyperthyroidism.

#### Personalized autophagosome vaccine

Autophagosome vaccine represents another approach to personalized vaccine therapy. A phase I trial investigated a novel autologous vaccine called DRibble vaccine, containing multiple specific tumor-associated antigens created from short-lived proteins (SLiPs) and defective ribosomal products (DRiPs) packaged in a double membrane microvesicle (NCT00850785) [65]. The major benefit of this strategy is to increase the presentation of TAAs that are not normally available for cross-presentation because they are short-lived. The study only enrolled 4 patients with stage IV NSCLC. DRibble vaccine was manufactured from malignant pleural effusions, given on days 14, 43, 57, 71, and 85, together with GM-CSF. Of the three evaluable patients, two had immune response against its antigen that is unique to the individual. However, all patients came off the study before completing the whole vaccination schedule due to disease progression. Researchers are exploring combinations of DRibble vaccines with T-cell agonists or ICIs to improve clinical responses. A major challenge remains low patient accrual, driven by poor performance status and long manufacturing times in the context of rapidly progressing disease.

#### Personalized peptide vaccine

NEO-PV-01, a personalized neoantigen peptide vaccine, was evaluated as first-line therapy in metastatic non-squamous NSCLC in combination with pembrolizumab and chemotherapy (NCT03380871). A total of 38 patients received the vaccine. The ORR was 69%, with only one reported severe adverse event (SAE) [66]. All vaccinated patients exhibited interferon-gamma (IFN-γ) secretion eight weeks post-vaccination. Neoantigen-specific immune responses were observed in 55% of patients, with 39% of epitopes eliciting CD4 + T cell responses and 31% eliciting CD8 + T cell responses. However, 45% of enrolled patients were unable to proceed to vaccination due to progressive disease, inadequate biopsy tissue, or insufficient mutational burden for high-quality neoantigen manufacture. Authors suggested using TMB to optimize candidate selection and switching to mRNA platforms to accelerate vaccine production.

A second phase Ib trial of NEO-PV-01 in combination with nivolumab was conducted in advanced solid tumor patients, including not only NSCLC patients but also melanoma and bladder cancer (NCT02897765) [67]. Among 82 patients, 27 had NSCLC. The immunological responses were similar or superior to the first trial. No treatment-related SAEs occurred. *De novo* neoantigen-specific CD4 + and CD8 + T cells were detected in all vaccinated patients, exhibiting memory phenotypes, cytotoxic potential, mutant specificity, durability, and tumor-homing ability. High TMB again correlated positively with immunogenicity. Clinical responses in NSCLC were comparable to historical anti-PD-1 monotherapy data, with an ORR of 39% in NSCLC, 59% in melanoma, and 27% in bladder cancer. Median PFS and OS in the NSCLC cohort were 8.5 months and 83% at 15 months follow-up, respectively.

Another personalized neoantigen vaccine, GEN-009, was investigated in combination with PD-1 inhibitors for patients with advanced solid tumors (NCT03633110). Preliminary data published in 2021 showed no SAEs [68]. Immune responses were persistent for at least 6 months in some patients, measured by robust IFN-γ secretion. However, there was no significant difference in PFS between responders and non-responders.

#### Personalized DC vaccine

A TP53-transfected dendritic cell-based vaccine (Ad.p53-DC) followed by chemotherapy was investigated in a randomized-controlled phase II trial in recurrent SCLC (NCT00617409). In this study, DCs were transfected with wild-type TP53 carried by an adenoviral vector. A total of 69 patients were enrolled and randomized into three arms: arm A (observation), arm B (vaccine alone), and arm C (vaccine plus all-trans-retinoic acid) [69]. The vaccine did not significantly improve ORR to salvage chemotherapy, with ORRs of 15.4%, 16.7%, and 23.8% for arms A, B, and C, respectively. No differences in OS were observed between arms. Immune responses, measured by peripheral blood interferon-γ secretion, were detected in 20% of patients in arm B and 43.3% in arm C. The investigators suggested that the negative survival results were partly due to a high dropout rate (20 patients) before vaccine administration, primarily because of clinical deterioration or withdrawal of consent. Despite these limitations, a “flattening” of the survival curve was observed in immune response-positive patients, with some surviving over five years. Building on this experience, Ad.p53-DC was further investigated in a single-arm phase II trial in relapsed SCLC in combination with dual ICIs ipilimumab and nivolumab (NCT03406715). Fourteen patients were enrolled, achieving a DCR of 42.85%. Median PFS was 63 days, and median OS was 120 days.

#### Personalized viral vector vaccine

Viral vectors are an attractive delivery platform for personalized cancer vaccines due to their inherent ability to stimulate cytotoxic T lymphocytes [70]. Additionally, they can encode large gene inserts, allowing the simultaneous targeting of multiple neoantigens in personalized vaccine strategies. NOUS-PEV is a viral vector-based personalized vaccine designed to target approximately 60 tumor-specific neoantigens. It is currently being evaluated in a phase Ib trial in combination with pembrolizumab in patients with unresectable stage III/IV melanoma and stage IV NSCLC (NCT04990479). Data published from the melanoma cohort reported no serious treatment-related adverse events [71]. Neoantigen-specific CD4 + and CD8 + T cell responses were detected in all evaluable patients. In patients who achieved a clinical response, expansion and diversification of vaccine-induced TCR clonotypes were observed, highlighting the ability of the viral vector platform to elicit robust and broad anti-tumor immunity.

### Mucosal vaccine

The mucosal immune system constitutes the most extensive peripheral immune network and serves as a frontline defense against microbial and dietary antigens. The respiratory tract represents the body’s second-largest mucosal surface area, following the gastrointestinal tract; consequently, mucosal immunity plays a critical role in the prevention and treatment of lung infections [72, 73]. Recent studies have shown that mucosal (intranasal) vaccination outperforms systemic intramuscular vaccination in controlling head and neck tumors, primary lung tumors, and lung metastases from breast cancer [74, 75]. A central mechanism underlying this enhanced efficacy of mucosal immunity is the robust induction of tissue-resident memory T cells (Trm) within the respiratory tracks and lung parenchyma, which can serve as a key determinant of vaccine response [76]. These Trm cells provide a new subset of long-lived CD8 + memory T cells. They differ from circulating memory T cells in their tissue location, gene expression, and function, providing long-term, rapid local protection against reinfection and cancer but can also cause autoimmune conditions by attacking healthy tissue. In addition, Trm cells are also predictive of response to anti–PD-1 therapy [77, 78]. Furthermore, intranasally delivered cancer vaccines can prevent tumor onset in spontaneous KRAS-mutant lung cancer mouse models [79]. However, this protection appears substantially diminished in metastatic settings, where disseminated tumors often arise outside the lung’s mucosal immune compartment. Further studies are needed to define the optimal clinical contexts and combinatorial strategies for effective mucosal vaccination.

## Immune adjuvants

**Fig. 5:**
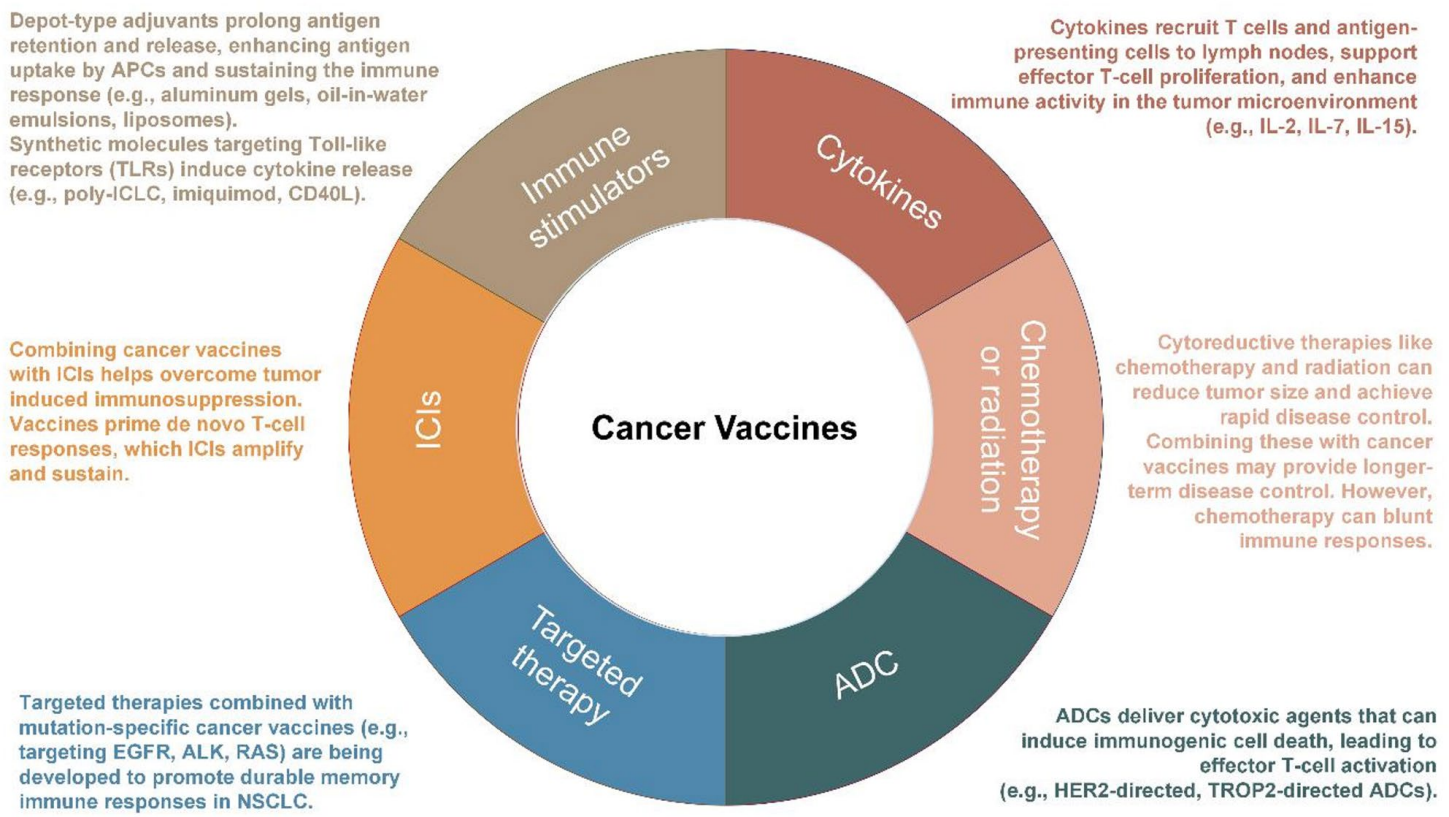
Vaccine Adjuvants. Laboratory-produced cancer vaccines often fail to fully activate the immune system. To enhance immunogenicity, small peptides or nonprotein antigens are commonly conjugated to larger carrier proteins and administered with adjuvants. Adjuvants can include immune stimulators, cytokines, chemotherapy or radiotherapy, antibody–drug conjugates, targeted therapies, and immune checkpoint inhibitors (ICIs). Together, these components enhance antigen presentation, promote dendritic cell activation, and drive a more potent and durable antitumor immune response. ADC, antibody–drug conjugate; ALK, anaplastic lymphoma kinase; APC, antigen-presenting cell; CD40L, CD40 ligand; EGFR, epidermal growth factor receptor; HER2, human epidermal growth factor receptor 2; ICI, immune checkpoint inhibitor; IL, interleukin; NSCLC, non-small cell lung cancer; poly-ICLC, polyinosinic–polycytidylic acid stabilized with poly-lysine and carboxymethylcellulose; RAS, rat sarcoma viral oncogene homolog; TLR, Toll-like receptor; TROP2, trophoblast cell surface antigen 2. Created with BioRender.com

Many laboratory-produced cancer vaccines, particularly peptide- and subunit-based formulations, lack sufficient intrinsic innate immune activation to induce robust antigen presentation and T-cell priming when administered alone. To elicit a potent immune response, small peptides or non-protein antigens typically require chemical conjugation to larger carrier proteins and co-administration with adjuvants. Adjuvants enhance vaccine immunogenicity through several mechanisms, including increasing antigen availability, activating innate immune pathways, and promoting the recruitment and maturation of antigen-presenting cells (APCs). Some formulations, including aluminum-based adjuvants, oil-in-water emulsions, and liposomal systems, can prolong antigen retention at the injection site, a phenomenon often referred to as a **“depot effect”**, thereby facilitating antigen uptake by APCs while also promoting local immune activation [80]. **Immunostimulatory adjuvants** enhance immune activation directly. Granulocyte-macrophage colony-stimulating factor (GM-CSF) is frequently employed; preclinical studies demonstrate that GM-CSF recruits and activates APCs at the injection site, enhancing T-cell responses and reducing tumor growth in both whole-tumor-cell and peptide-based vaccines [81]. Other cytokines, including IFN-α, IFN-γ, IL-2, IL-12, IL-15, IL-18, and IL-21, have also shown immunological efficacy when incorporated into vaccine adjuvant regimens [82]. **Adjuvants targeting Toll-like receptors (TLRs)** are particularly promising for cancer vaccines. For example, polyinosinic: polycytidylic acid [poly(I: C)] and polyinosinic-polycytidylic acid-lysine carboxymethylcellulose (poly-ICLC) are synthetic double-stranded RNA molecules that bind endosomal TLR3, mimicking viral infection and inducing type I interferon and pro-inflammatory cytokine production [83]. Imiquimod, a TLR7 agonist, is widely used in lung cancer vaccine studies; it activates TLR7/8, drives a Th1-type response, and promotes cytokine production [84]. Furthermore, TLR7/8 has been directly conjugated to charge-modified neoantigens to enhance uptake by and activation of APCs [85] (Fig. 5).

## Challenges and opportunities in lung cancer vaccine

### Optimal disease setting for cancer vaccines

Outside the lung cancer, cancer vaccines are generally more effective when administered in the adjuvant setting, following definitive surgery or other curative-intent therapy, than in the metastatic setting [86, 87]. Several biological and immunological principles support this paradigm. First, following resection of early-stage NSCLC, patients typically have either no or low tumor burden, i.e., minimal residual disease (MRD). In this context, the immune-mediated eradication of residual tumors in micrometastases is more effective than targeting bulky or disseminated metastatic lesions [88]. Second, the adjuvant setting is characterized by an absent or minimal presence of immunosuppressive TME, with low level of regulatory T cells, MDSCs, and suppressive cytokines in the immunosuppressive TME. This environment allows vaccines to more effectively prime, expand, and differentiate tumor-specific T cells capable of eliminating residual disease [89, 90]. In contrast, metastatic lung cancer is marked by chronic antigen exposure, which drives profound T-cell exhaustion and diminishes cytotoxic function, proliferative capacity, and responsiveness to vaccination [91]. Third, in the adjuvant setting, vaccines can be administered alongside or after treatments that enhance antigen release (e.g., surgery, radiation, some chemotherapies), providing a natural boost to vaccine-induced responses. Conversely, patients with metastatic disease often receive therapies that impair immune function, further limiting vaccine efficacy. Fourth, early-stage lung cancers harbor fewer genomic mutations and immune-evasion mechanisms than metastatic lesions, which frequently downregulate antigen presentation pathways or disrupt interferon signaling to escape immune surveillance [92]. Fifty, clinical experience with ICIs in NSCLC further supports this framework: ICIs consistently demonstrate greater benefit in early-stage and perioperative settings than in metastatic disease [86]. Finally, a recent study demonstrated that SARS-CoV-2 mRNA vaccines can sensitize tumors to ICIs, even in immunologically “cold” cancers, highlighting the potential of broadly applicable, off-the-shelf immunomodulatory strategies to overcome key barriers such as low immunogenicity, immune suppression, and limited T-cell infiltration [93]. If validated in prospective clinical trials, this approach could rapidly transform standard-of-care immunotherapy by enhancing both its effectiveness and accessibility. Together, these findings indicate that the adjuvant setting provides a uniquely favorable immunological window in which cancer vaccines are more likely to induce durable, protective antitumor immunity, reduce recurrence risk, and improve long-term outcomes in lung cancer. In the metastatic setting, cancer vaccines alone have modest clinical antitumor effect and are frequently given in combination with effective therapeutics for a rapid, robust, and effective antitumor effects needed to control the cancer.”

### Mechanistic constraints on immunotherapy in oncogene-driven NSCLC

ICI therapy has shown limited efficacy in oncogene-driven NSCLC, with no significant survival benefit observed in patients with EGFR-mutated tumors [94]. Most oncogene-driven NSCLC, with the exception of KRAS-mutant and BRAF V600E-mutant subtypes, tend to have low TMB, resulting in reduced immunogenicity [48, 49, 95, 96]. Consequently, small molecule TKIs remain the standard first-line treatment for EGFR-mutated and ALK-rearranged NSCLC. However, resistance to TKIs is almost universally inevitable [97, 98]. Preclinical studies suggest that the poor response to ICIs in ALK-driven NSCLC may be due to inadequate CD8 + T cell priming against ALK antigens. Interestingly, this limitation could potentially be overcome by targeted vaccination strategies aimed at restoring tumor immunogenicity [53]. Nonetheless, the feasibility of personalized cancer vaccines in oncogene-driven NSCLC remains uncertain. A recent personalized vaccine trial in pancreatic cancer, another low-TMB tumor type, revealed that low TMB was associated with poor vaccine efficacy and significant manufacturing challenges.

Accordingly, the application of personalized vaccine strategies in oncogene-driven NSCLC presents several unresolved obstacles. Optimal combination approaches incorporating oncogenic peptides have not yet been defined. Ongoing trials are evaluating ALK peptide vaccines in combination with ALK inhibitors as front-line therapy, notably without concurrent ICIs. In parallel, CIMAvax-EGF is being investigated in combination with ICIs following the development of acquired TKI resistance (NCT05950139; NCT06095934).

### Mechanistic determinants of effective neoantigen selection

The limited clinical success of cancer vaccines is partly attributable to suboptimal neoantigen selection strategies. McGranahan and colleagues have outlined several key principles for effective neoantigen selection: (1) **Clonality**: Neoantigens should be *clonal*, meaning they are present in a large proportion of cancer cells. This helps prevent immune evasion through T cell–mediated selection against subclonal neoantigens. (2) **Distinction to Self**: Neoantigens should be highly distinct from self or known antigens to avoid immune tolerance and enhance immunogenicity. (3) **Antigenicity**: Neoantigens must be both transcribed and translated to effectively stimulate an immune response. (4) **Genomic Stability**: Neoantigens should arise in regions with a low likelihood of deletion or alteration during tumor evolution, which can be estimated based on their genomic context. (5) **Broad HLA Binding**: Neoantigens capable of binding to multiple HLA alleles are preferred to reduce the risk of immune resistance due to HLA loss [99]. In addition, some TAAs harbor epitopes shared with other human proteins expressed in normal tissues, which may increase the risk of **off-target T-cell reactivity** and compromise both vaccine safety and efficacy. Accordingly, rigorous epitope filtering strategies to exclude shared sequences within TAAs have been proposed as an important consideration in neoantigen selection [100].

Accurately identifying neoantigens that are both presented by MHC molecules and capable of inducing an immune response remains a major challenge [101]. This process depends not only on peptide–MHC binding affinity but also on the subsequent immunogenicity of the bound peptide [102]. The most commonly used approach involves in silico prediction of binding affinities between thousands of peptides and a limited subset of MHC class I alleles [103, 104]. However, these methods often oversimplify the complexity of antigen presentation. Indeed, in vivo studies have shown that fewer than 6% of predicted neoantigens elicit a functional T-cell response, and even peptides with low predicted affinity can be immunogenic [105, 106]. Alternative strategies include direct identification of mutated peptides presented on MHC molecules from primary tumor tissue, though this technique is limited by low sensitivity. Another approach involves isolating neoantigens that have already triggered a T cell response, though this is restricted to pre-existing immune reactivity [105, 107]. Additionally, prioritizing neoantigens that emerge before a whole-genome doubling (WGD) event may enhance vaccine efficacy, as neoantigens arising post-WGD are more likely to be lost during tumor progression [54]. Refining these selection methods is critical to enhance the immunogenicity and clinical efficacy of personalized cancer vaccines.

### The role of HLA haplotypes in determining immune responses

**Fig. 6:**
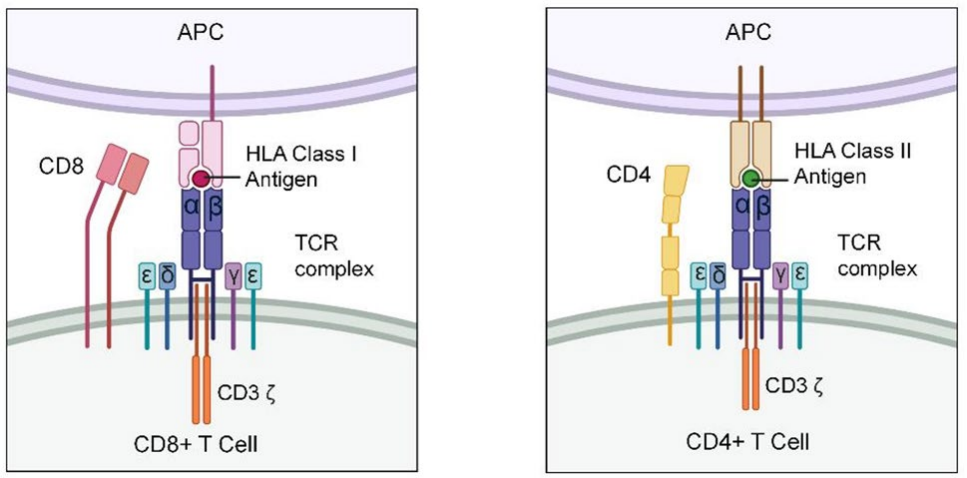
Role of HLA in Antigen Presentation. (**A**) HLA Class I molecules primarily present intracellular peptides to CD8 + cytotoxic T cells, initiating targeted tumor cell killing. (**B**) HLA Class II molecules present extracellular peptides to CD4 + helper T cells, supporting CD8 + T cell activation, B cell maturation, and antibody production. Together, these pathways orchestrate adaptive immune responses critical for effective antitumor immunity. APC, antigen presenting cell; TCR, T cell receptor. Created with BioRender.com

Host genetic variation in HLA haplotypes plays a crucial role in shaping antigen presentation, defining immune responses, generating autoantibodies during cancer immunotherapy, and determining the association with irAEs [108, 109]. HLA Class I molecules (HLA-A, -B, and -C) primarily present intracellular peptides to CD8 + T cells, whereas HLA Class II molecules (HLA-DR, -DQ, and -DP) present extracellular peptides to CD4 + T cells (Fig. 6). The loss of expression of HLA molecules and molecules related to antigen processing has been contributing to the low efficacy of therapeutic cancer vaccine in the metastatic disease. 30% of NSCLCs show selective downregulation on HLA-I tumor cells (one or more β2M, HLA-A, HLA-B/C subunits) [110–114]. HLA loss can arise through multiple mechanisms, including irreversible genetic alterations that may require gene-editing approaches for restoration, as well as more common reversible transcriptional, post-transcriptional, or epigenetic changes that can potentially be restored through pharmacologic interventions such as interferon signaling or epigenetic modulation [113, 115, 116]. Furthermore, better quality of antigen presentation in adjuvant setting than the metastatic setting: After tumor removal, antigen-presenting cells can process tumor antigens released during surgery or minimal residual disease in a context that favors activation rather than suppression. In metastatic disease, chronic exposure to tumor antigens leads to T-cell exhaustion and suboptimal antigen presentation. There are unmet needs to incorporate empirical HLA class I immunopeptidomics into neoantigen selection pipelines, as computationally predicted binders do not always correspond to peptides that are naturally processed and presented on tumor cells [117]. However, this is challenging due to the diverse pool of HLA haplotypes that vary to age, race and ethnicity.

### Immune activation does not necessarily predict clinical benefit

Although neoantigens hold strong potential as targets for T cell-mediated immune responses, immune activation does not consistently translate into clinical benefit. Higher neoantigen loads are generally associated with improved outcomes, and neoantigen-targeted therapies have demonstrated tumor-killing activity in both preclinical and clinical models. Nonetheless, many cancer vaccine trials have reported underwhelming results. Despite eliciting robust immune responses, many of these trials report limited or no improvements in clinical outcomes.

**Fig. 7:**
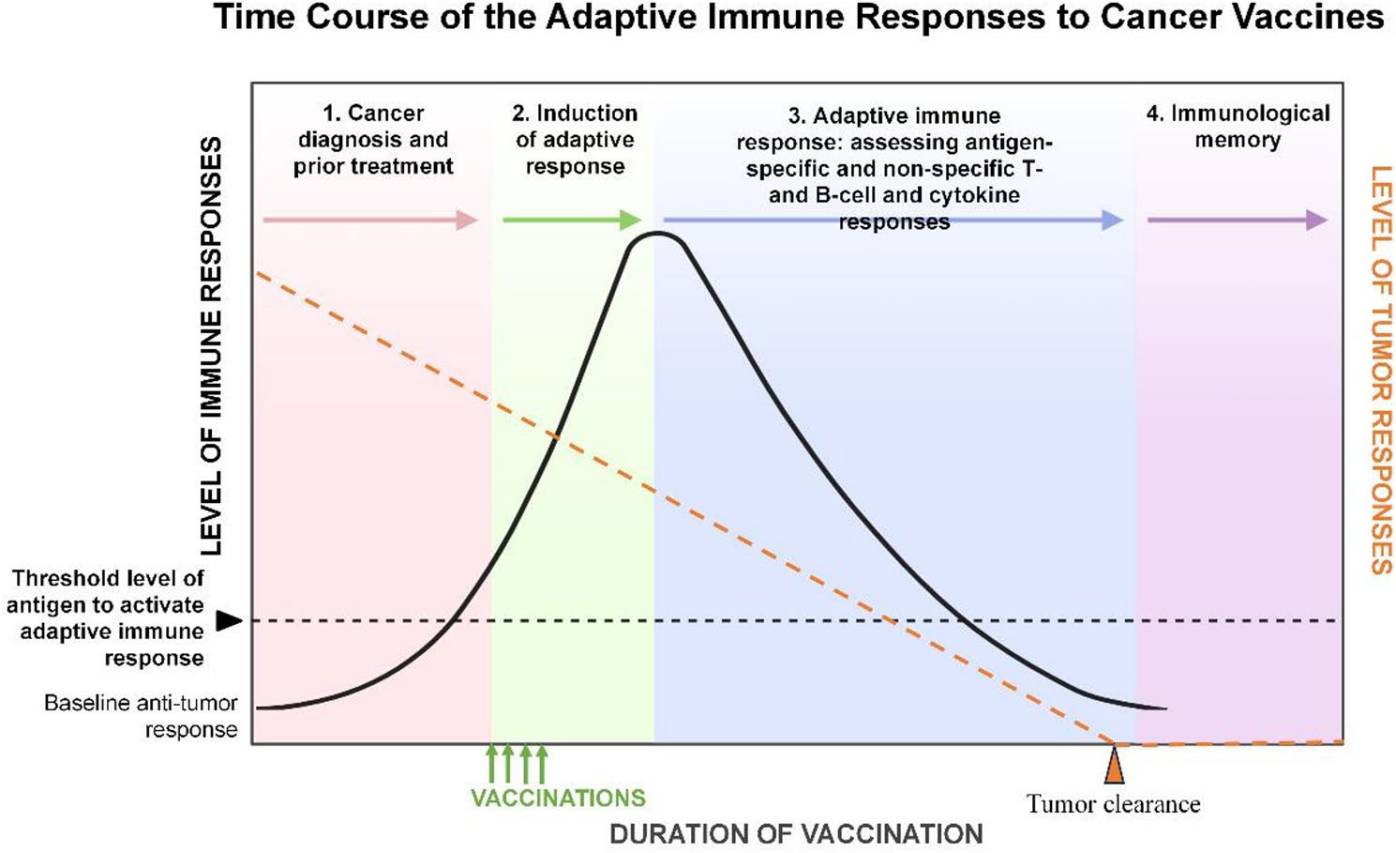
Time Course of the Adaptive Immune Responses to Cancer Vaccines. Currently, there is no universally accepted standard exists for assessing vaccine-induced immunogenicity. Both surrogate markers and timing of evaluations vary substantially across studies. Traditional assays measure cytokine production and quantify T-cell frequencies, but these approaches often lack specificity. More advanced techniques detect antigen-specific antibodies and monitor T-cell receptor (TCR) clonotypes, while functional assays evaluate T-cell trafficking to tumor sites and assess cytotoxic potential. Longitudinal monitoring using TCR repertoire sequencing allows the tracking of clonal dynamics and the persistence of central memory T cells, providing deeper insight into the durability of vaccine-induced immune responses. TAA, tumor-associated antigen; TSA, tumor-specific antigen; TCR, T-cell receptor. Created with BioRender.com

Currently, there is no universally accepted gold standard for measuring vaccine-induced immunogenicity. Surrogate markers and the timing of immune assessments vary widely across studies. Traditional approaches often measure cytokine production (e.g., IFN-γ, TNF-α, IL-2) or quantify T cell frequencies [66, 118, 119]. While informative, these metrics frequently lack specificity for vaccine-induced responses and may not accurately reflect functional antitumor activity. More recent studies aim to improve specificity by identifying neoantigen-specific antibodies or T cells. Although these markers offer greater precision, they still fail to capture the full complexity and functional dynamics of the immune response. For example, central memory T cells support durable immunity through long-term persistence and recall capacity, while cytotoxic CD4⁺ T cells are increasingly recognized as important contributors to antitumor immune responses [120–123]. Figure 7 illustrates the time course of the adaptive immune responses to cancer vaccines.

Functional assays are being employed to evaluate T-cell trafficking and cytotoxic potential at tumor sites, providing more detailed insights into immune activity. However, these methods capture only a fraction of the broader immune response. The immune system is a highly complex, multilayered network, and accurately quantifying its components remains a significant challenge. Practical limitations further complicate these efforts, including the limited availability of patient-derived samples and the need for sophisticated bioinformatics and high-dimensional data integration. These barriers continue to hinder the establishment of standardized, universally accepted metrics for evaluating vaccine-induced immunity.

### Vaccine timing and duration

Similar to ongoing debates regarding the optimal duration of ICI therapy, whether treatment should be limited to 1–2 years or continued indefinitely, the ideal timing and duration of cancer vaccine administration remain unresolved [124]. Vaccine regimens vary considerably across clinical trials, typically involving three to ten total doses. These are often coordinated with concurrent chemotherapy or ICI therapies to reduce patient burden and streamline logistics.

Another critical consideration is the choice of prime-boost strategy. Most clinical studies employ a homologous prime-boost approach, repeatedly delivering the same immunogen to amplify the pool of memory B and T cells generated by the initial dose [70, 125]. However, repeated administration of the same vaccine may lead to diminished cellular responses over time, largely due to the formation of neutralizing antibodies against the vaccine vector or its components [126]. To overcome this limitation, *heterologous prime-boost* strategies, using different vaccine platforms or vectors for priming and boosting, have been explored. These approaches have demonstrated robust and durable immune responses in several viral vector-based trials, although their superiority over homologous strategies has not been definitively established [71]. Finally, the sequencing of cancer vaccines and ICIs is under active investigation. Optimizing timing to permit effective CD8 + T cell priming while maintaining long-term memory responses may be critical to overcoming resistance and enhancing the durability of antitumor immunity [88, 127, 128].

### Limitations of vaccine monotherapy and the need for combination strategies

Despite strong scientific rationale, therapeutic cancer vaccines have historically shown modest immunogenicity and limited clinical benefit in lung cancer. Several intrinsic biological and immunological barriers limiting the efficacy of lung cancer vaccines that have historically achieved limited clinical activity. Overcoming these foundational obstacles will require combination strategies that remodel the TME, boost antigen presentation, and enhance T-cell infiltration and functionality. To date, the most widely explored approach has been the combination of cancer vaccines with ICIs, particularly in personalized treatment settings [129, 130].

Beyond anti–PD-(L)1 based combinations, additional strategies targeting key immunosuppressive pathways within the TME may further augment vaccine efficacy in lung cancer. For example, approaches aimed at reducing regulatory T cells, such as low-dose cyclophosphamide, anti-CD25 antibodies, or CCR4 antagonists, can mitigate Treg-mediated suppression and improve the expansion and function of vaccine-induced effector T cells [131]. In parallel, therapies directed against MDSCs, including CXCR2 inhibition, arginase or iNOS blockade, and PI3Kγ inhibitors, may alleviate myeloid-driven suppression and facilitate T-cell migration into lung tumors [132, 133]. Targeting the HGF/MET axis represents another promising avenue, as MET activation promotes immune exclusion, tumor invasiveness, and resistance to immunotherapy; MET inhibitors may therefore potentiate vaccine-induced infiltration and cytotoxicity [134]. Similarly, CSF1/CSF1R blockade can reprogram tumor-associated macrophages from an immunosuppressive, M2-like phenotype toward a pro-inflammatory state that enhances antigen presentation and supports vaccine-primed T-cell responses [135]. Inhibition of the Wnt/β-catenin pathway, which is frequently implicated in “immune-cold” lung tumors lacking T-cell infiltration, may further improve responsiveness by converting non-inflamed tumors into more permissive, T-cell–accessible TME [136]. Together, these combinational strategies underscore the opportunity to combine cancer vaccines with agents that remodel the suppressive lung TME, thereby amplifying T-cell priming, improving infiltration into tumor sites, and ultimately enhancing the depth and durability of anti-tumor immunity.

## Summary and future perspectives

Cancer vaccines are rapidly evolving, with lung cancer representing a major area of ongoing translational and clinical investigation. Current clinical trials increasingly focus on combining vaccines with ICIs and other immunotherapies to enhance antitumor efficacy (Tables 9 and 10). Despite encouraging progress, several key challenges persist, including the identification of truly immunogenic neoantigens, optimization of vaccine delivery platforms and delivery strategies, and the rational integration of complementary therapies to overcome resistance to ICIs. Future vaccine development must carefully consider multiple factors, including antigen selection, vaccine formulation, choice of adjuvants, delivery platforms, dosing schedules, treatment duration, and optimal sequencing with other therapeutic modalities. Technological innovations, such as improved neoantigen prediction algorithms, enhanced HLA-binding analyses, and more sensitive immune monitoring, together with a deeper understanding of tumor biology and cancer immunology, are accelerating the development of next-generation vaccines. These advances highlight the potential of cancer vaccines for both therapeutic and preventive applications by inducing durable, tumor-specific immune responses, counteracting immune evasion, and ultimately improving patient outcomes. Continued clinical investigation will be critical to define optimal vaccination strategies, validate predictive biomarkers, and translate mechanistic and immunological insights into meaningful clinical benefit for patients with lung cancer.

**Table 9:** Highlight key lung cancer vaccine trials targeting oncogenic mutations

Cancer target	Cancer type	Vaccine type	NCT number	Disease setting	Eligibility criteria	Trial status	Study phase	No. patients	Study period	Combination	Vaccine name
**EGFR**	NSCLC	Peptide	NCT04298606	Stage IA-IIIA (prevention and adjuvant)	(A) High risk for lung cancer subjects; (B) Lung cancer survivors > 3 months post-treatment.	Ongoing	I	N/A	2021-11 to 2024-11	None	CIMAvax
	NSCLC, squamous H&N cancer	Peptide	NCT02955290	Stage IV	Eligible for nivolumab standard-of-care	Active	I/II	23	2016-12 to 2027-12	ICI	CIMAvax
	EGFR + NSCLC	Peptide	NCT06095934	Stage IIIB-IV (EGFR-TKI resistance)	EGFR-mutant NSCLC with disease progression on EGFR-TKI	Ongoing	I/II	N/A	2022-03 to 2025-12	ICI and chemotherapy	N/A
**ALK**	ALK + NSCLC	Peptide	NCT05950139	Stage IV	ALK-positive NSCLC with stable disease on ALK inhibitor therapy	Ongoing	I/II	N/A	2024-05 to 2029-07	ALK inhibitors	N/A
**Multiple RAS and CTNNB**	Solid tumors (pancreatic cancer, NSCLC, and CRC)	Viral vector and mRNA	NCT03953235	Stage IV	RAS or CTNNB1-mutated NSCLC previously treated with first-line chemo-immunotherapy	Completed	I/II	19	2019-07 to 2023-03	ICI	GRT-C903 /GRT-R904
**KRAS**	KRAS + solid tumors (G12D or KRAS G12V	Peptide	NCT06253520	Stage IV	KRAS-mutant solid tumors recurring after standard therapy	Ongoing	I	N/A	2024-05 to 2033-06	CART and IL2	GRT-C903 /GRT-R904
	KRAS/NRAS + solid tumors (G12D or G12R)	Oligonucleotide and peptide	NCT04853017	Stage IV	Positive ctDNA and/or elevated tumor biomarker after standard therapy	Ongoing	I	N/A	2021-10 to 2026-03	none	ELI-002
	KRAS + NSCLC and pancreatic cancer	Peptide	NCT06015724	Stage IV	KRAS-mutant NSCLC progressed on non-KRAS therapy	Ongoing	II	N/A	2024-01 to 2026-01	Daratumumab and ICI	Targovax TG-01/Stimulon QS-21
	KRAS + NSCLC (KRAS G12C, KRAS G12V, KRAS G12D, KRAS G12A, KRAS G13D or KRAS G12R)	Peptide	NCT05254184	Unresectable stage III-IV	KRAS-mutant NSCLC: treatment-naïve stage IV or previously treated stage III	Ongoing	I	N/A	2022-11 to 2027-04	ICI	N/A
	KRAS + solid tumors (G12D, G12V, G13D or G12C)	mRNA	NCT03948763	Stage IV	KRAS-mutant solid tumors after exhaustion/intolerance/ineligibility for standard options	Completed	I	N/A	2019-06 to 2022-08	ICI	mRNA-5671/V941

EGFR, epidermal growth factor receptor; NSCLC, non-small cell lung cancer; H&N, head and neck; ICI, immune checkpoint inhibitor; TKI, tyrosine kinase inhibitor; ALK, anaplastic lymphoma kinase; CRC, colorectal cancer; CTNNB, catenin beta-1 (β-catenin); mRNA, messenger ribonucleic acid; CART, chimeric antigen receptor T-cell therapy; IL2, interleukin-2

**Table 10:** Highlights personalized vaccine trials

Cancer type	Vaccine type	NCT number	Disease setting	Trial status	Study phase	Eligibility criteria	No. patients	Study period	Combination	Vaccine name
**Personalized neoantigen vaccine (peptide)**	NSCLC	NCT00098085	Stage IB-IIIA (adjuvant)	Completed	II	Planned surgical resection; no prior therapy	4	2003-09 to 2007-11	N/A	Heat-shock protein peptide complex-96 (HSPPC-96)
	NSCLC	NCT03380871	Unresectable or metastatic	Completed	I	No prior immunotherapy	38	2018-05 to 2021-02	Chemotherapy, ICI, poly-ICLC	NEO-PV-01 vaccine
	NSCLC, melanoma and bladder cancer	NCT02897765	Unresectable or metastatic	Completed	I	Immunotherapy naïve	82	2016-10 to 2020-05	ICI, poly-ICLC	NEO-PV-01 vaccine
	Solid tumors	NCT03633110	Stage IV	Completed	I/II	mNSCLC starting first line immunotherapy	16	2018-08 to 2022-02	ICI	GEN-009 vaccination
	Solid tumors	NCT05269381	Advanced	Ongoing	I/II	Eligible for pembrolizumab	N/A	2022-03 to 2026-02	ICI, GM-CSF and chemotherapy	PNeoVCA
**Personalized DNA vaccine**	Solid tumors	NCT03548467	Locally advanced or metastatic	Completed	I/II	Must have received ICI	N/A	2018-04 to 2023-01	bempegaldesleukin (NKTR-214)	VB10.NEO
**Personalized mRNA vaccine**	Solid tumors	NCT03639714	Stage IV	Completed	I/II	NSCLC planning platinum chemotherapy	15	2019-02 to 2022-11	ICI	GRT-C901/GRT-R902
	NSCLC	NCT06077760	Resected Stage II-IIIB	Ongoing	III	No prior systemic therapy; disease-free post-surgery	N/A	2023-12 to 2035-12	ICI	mRNA-4157 (V940)
	NSCLC	NCT06623422	Resectable stage II-IIIB	Ongoing	III	Without pCR after neoadjuvant chemo-immunotherapy and surgery	N/A	2024-10 to 2033-05	ICI	
	Solid tumors	NCT03313778	Adjuvant NSCLC	Ongoing	I	Resectable NSCLC	N/A	2017-08 to 2026-06	ICI	
	Solid tumors	NCT03289962	Locally advanced or metastatic	Ongoing	I	Progressed after standard therapy.	N/A	2017-12 to 2025-03	ICI	autogene cevumeran (RO7198457)
	Melanoma and NSCLC	NCT04990479	Stage IV NSCLC	Ongoing	I	PD-L1 ≥ 50%; no prior systemic therapy	N/A	2021-06 to 2024-10	ICI	NOUS-PEV
**Personalized DC vaccine**	SCLC	NCT00617409	Extensive stage	Completed	II	Non-progressive disease after first-line therapy	69	2007-10 to 2019-01	Chemotherapy and all-trans retinoic acid	Ad.p53-DC
**Tumor-derived autophagosome vaccine**	NSCLC	NCT03057340	Stage IIIA/IIIB (adjuvant)	Unknown	I	Progression after chemoradiation	N/A	2017-06 to 2020-12	GM-CSF or imiquimod	Dribbles
**Personalized viral vector**	Melanoma and NSCLC	NCT04990479	Stage IV NSCLC	Ongoing	I	PD-L1 ≥ 50%; no brain metastases	N/A	2021-06 to 2024-03	ICI	NOUS-PEV

ICI, immune checkpoint inhibitor; NSCLC, non-small cell lung cancer; SCLC, small cell lung cancer; ctDNA, circulating tumor DNA; DC, dendritic cell; DNA, deoxyribonucleic acid; mRNA, messenger ribonucleic acid; GM-CSF, granulocyte-macrophage colony-stimulating factor; HSPPC-96, heat-shock protein peptide complex-96; pCR, pathological complete response; poly-ICLC, polyinosinic-polycytidylic acid stabilized with poly-L-lysine and carboxymethylcellulose; NKTR-214, bempegaldesleukin; Ad.p53-DC, adenovirus-p53 dendritic cell vaccine

## Data Availability

No datasets were generated or analysed during the current study.

## Abbreviations

AAH: Atypical adenomatous hyperplasia
ADC: Antibody-drug conjugate
ADCC: Antibody-dependent cell cytotoxicity
AE: Adverse event
AIS: Adenocarcinoma in situ
ALK: Anaplastic lymphoma kinase
APC: Antigen-presenting cell
BCG: Bacillus Calmette-Guérin
BCR: B-cell receptor
BiTEs: Bispecific T-cell engagers
CAR-T: Chimeric antigen receptor T cells
CD40L: CD40 ligand
CDKN2A: Cyclin dependent kinase inhibitor 2 A
CEA: Carcinoembryonic antigen
ChAd68: Chimpanzee adenovirus serotype 68
CR: Complete response
CTL: Cytotoxic T lymphocyte
CTNNB: Catenin beta
DC: Dendritic cells
DCR: Disease control rate
DFS: Disease-free survival
DLT: Dose limiting toxicity
DRiPs: Defective ribosomal products
DVT: Deep vein thrombosis
EBV: Epstein–Barr virus
EGFR: Epidermal growth factor receptor
FDA: U.S. Food and Drug Administration
GM-CSF: Granulocyte-macrophage colony-stimulating factor
HBV: Hepatitis B virus
HCV: Hepatitis C virus
HER2: Human epidermal growth factor receptor 2
HLA: Human leukocyte antigen
HLAp: HLA-presented peptides
HPV: Human papillomavirus
ICIs: Immune checkpoint inhibitors
IFN-γ: Interferon-gamma
IL: Interleukin
IL-2: Interleukin-2
ISCOMATRIX: Immune-stimulating complex matrix (adjuvant platform)
KRAS: Kirsten rat sarcoma viral oncogene homolog
LFA: Lymphocyte function-associated antigen
LUAD: Lung adenocarcinoma
MAGE: Melanoma-associated antigen
MDSCs: Myeloid-derived suppressor cells
MHC: Major histocompatibility complex
MIA: Minimally invasive adenocarcinoma
mos: Minimally invasive adenocarcinoma Months
mOS: Median overall survival
mPFS: Median progression free survival
MRD: Minimal Residual Disease
MTD: Maximum tolerated dose
MUC1: Mucin 1
NGS: Next-generation sequencing
NK cells: Natural killer cells
NSCLC: Non-small cell lung cancer
NY-ESO-1: New York esophageal squamous cell carcinoma 1
ORR: Objective response rate
OS: Overall survival
pCR: Pathological complete response
PD: Progressive disease
PD-1: Programmed cell death protein 1
PD-L1: Programmed cell death ligand 1
PFS: Progression-free survival
PM2.5: Particulate matter less than 2.5 microns
Poly-ICLC: Polyinosinic-polycytidylic acid-lysine carboxymethylcellulose
PR: Partial response
PTEN: Phosphatase and tensin homolog
RB1: RB transcriptional corepressor 1
samRNA: Self-amplifying mRNA
SCLC: Small cell lung cancer
SD: Stable disease
SLiPs: Short-lived proteins
SMARCA4-UT: SMARCA4-deficient undifferentiated tumor
TAA: Tumor-associated antigen
TAF: Tumor-associated fibroblast
TAMs: Tumor-associated macrophages
TCR: T-cell receptor
TEIPP: T-cell Epitopes Associated with Impaired Peptide Processing
TILs: Tumor-infiltrating lymphocytes
TKIs: Tyrosine kinase inhibitors
TLR: Toll-like receptor
TMB: Tumor mutation burden
TME: Tumor microenvironment
TP53: Tumor protein p53
TRAEs: Treatment-related adverse events
Treg: Regulatory T cell
TRICOM: Three co-stimulatory molecules (B7-1, ICAM-1, and LFA-3)
TRM: Tissue-resident memory T cells
TROP2: Trophoblast cell surface antigen 2
TSA: Tumor-specific antigen
WGD: Whole-genome doubling
WT1: Wilms tumor gene 1
